# Allometry of Ingestion Among Habitat Mimicking Praying Mantises

**DOI:** 10.1002/ece3.73091

**Published:** 2026-02-13

**Authors:** Christopher Oufiero, Marlena Wood, Elizabeth McMillan

**Affiliations:** ^1^ Department of Biological Sciences Towson University Towson Maryland USA

**Keywords:** consumption, feeding, functional response, Mantodea, predation

## Abstract

Ingestion is the process of consuming a resource and is a component of an organism's handling time, which can limit the acquisition of additional resources and decrease predation rate. If a predator spends more time handling prey, it will have less time to seek out additional prey. Variation in ingestion may therefore impact energy fluxes and ecosystem stability. Body size has been proposed to affect ingestion, with larger organisms predicted to have a reduced handling time, potentially scaling like metabolic rate. The goal of this study was to examine the allometric relationships of ingestion, a proxy for handling time, among praying mantis species with different camouflage strategies in a phylogenetic context. We measured the time it takes adult female mantises to ingest a standard prey using time‐lapse photography in 1–8 individuals among 14 species of Mantodea 3–5 times, resulting in 324 trials from 66 individuals. We examined the scaling of ingestion in relation to mantis body size using both a phylogenetic general linear mixed model to account for within‐species variation, as well as a phylogenetic generalized least‐squares approach on species means. We also compared the scaling of our praying mantis ingestion allometry to other allometric relations of other organisms from a larger database (e.g., other insects, arachnids, and vertebrates). We found that the ingestion rate and time scale with a power‐law function regardless of the camouflage strategy. We also found that mantis prey‐mass‐specific ingestion scaled more like arachnids than insects when compared to the larger database. Comparing our scaling of ingestion to published values of metabolic scaling, we found a higher slope for ingestion. Together, our results suggest that larger praying mantises can ingest more resources than may be needed based on their metabolic rate, which may influence their role in the ecosystem.

## Graphical abstract

We investigate the allometry of ingestion among habitat mimicking praying mantises. We found strong relations to body size, but not camouflage. We compare our results to handling time in other predatory groups.

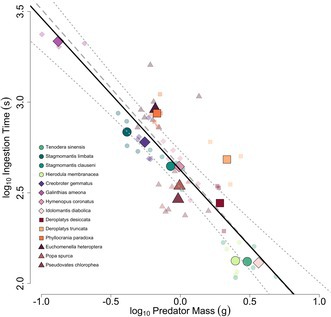

## 1 Introduction

One of the most important components of a predator's functional response is its handling time, which includes the time needed to attack, subdue, ingest, and digest a prey (Charnov 1976; Coblentz and DeLong 2021; Hassell et al. 1976; Holling 1966; Jeschke et al. 2002; Papanikolaou et al. 2021; Sentis et al. 2013). Of these components, the time to eat (i.e., ingestion) is one of the more commonly measured metrics among animals (Andrews et al. 1987; Coblentz et al. 2022; Cruz‐Neto et al. 2001; Shipley et al. 1994; Spitze 1985; Uiterwaal et al. 2022). The ingestion of resources links the process of attacking and subduing a resource to its digestion and has been modeled as a separate component of handling time in functional responses (Jeschke et al. 2002; Novak 2023; Novak et al. 2025; Papanikolaou et al. 2021). Ingestion can last much longer than attacking prey, but is typically less than digesting it. For example, it takes praying mantises 50–100 ms to capture a prey item once the attempt has been initiated, but up to 96 h to fully digest, with the ingestion lasting seconds to minutes (Corrette 1990; Holling 1966; McCue et al. 2016; Oufiero et al. 2016, 2024; Oufiero 2022). Ingestion time can limit the consumer's ability to acquire additional resources and may make them susceptible to predation. As many species can still obtain a resource while digesting, measuring just the time it takes to ingest the prey may be a good representation of the time a consumer cannot acquire additional resources as the entrance to the gastrointestinal tract is occupied by the current process of ingesting (Coblentz et al. 2022; Jeschke et al. 2002; Novak 2023; Novak et al. 2025). Variation in ingestion affects the functional response of a consumer as well as predator–prey dynamics. For example, an increase in ingestion will increase handling time, which can decrease the overall predation rate as the predator is spending more time processing the current resource as opposed to trying to obtain an additional resource (Aljetlawi et al. 2004; Holling 1966; Jeschke et al. 2002).

Ingestion can be affected by several biotic and abiotic factors, such as size, morphology, temperature, and prey characteristics (Andrews et al. 1987; Coblentz et al. 2022; Cruz‐Neto et al. 2001; Iles 2014; Shipley et al. 1994; Spitze 1985; Uiterwaal and DeLong 2020; Verwaijen et al. 2002). As many biological rates may be related (Glazier 2015; Hatton et al. 2019; Lindstedt and Hoppeler 2023), handling time, including the time to ingest, has been proposed to scale similarly to metabolic rate, especially since the two rates are associated with energy fluxes (Brose 2010; Brose et al. 2008; Vucic‐Pestic et al. 2010). The size of the consumer has been predicted to affect ingestion as larger organisms should have a reduced ingestion time, similar to the scaling of other biological features (Brose 2010; Brose et al. 2008; Coblentz et al. 2025; Uiterwaal and DeLong 2020). However, experimental evidence suggests otherwise for the similarity in the scaling of handling time and metabolic rate, with different scaling for the two rates (Rall et al. 2012; Vucic‐Pestic et al. 2010), potentially due to morphological constraints on the feeding apparatus (Andrews et al. 1987; Mcgee et al. 2015; Vucic‐Pestic et al. 2010). Morphology may also play a crucial role as feeding appendages have evolved and diversified to acquire different types of resources (e.g., teeth, mandibles, and cranial morphology). The variation in these structures may all affect the ingestion rate and reflect dietary selection to process those items in a more efficient manner (Felice et al. 2019; Law et al. 2018). Examining the allometric scaling of ingestion across species may provide information on how the evolution of differently sized predators might affect the time to handle prey and thus the functional responses of varying sized predators (Cruz‐Neto et al. 2001; Cundall and Deufel 2006; Cuthbert et al. 2020; Iles 2014; Kisdi and Liu 2006; Shipley et al. 1994; Vucic‐Pestic et al. 2010). Examining the allometric scaling of ingestion among a group of predators within a phylogenetic context can also provide information on the evolution of species that may adaptively vary from this scaling, suggesting selection for morphological features that increase or decrease ingestion or species that fill different ecological niches (Lindstedt and Hoppeler 2023). These relationships might also provide information on the energy fluxes of ecosystems among different‐sized predators and their relation to metabolic rate (Brose et al. 2008; Coblentz et al. 2025). However, few studies have examined ingestion across diverse species, spanning a range of body sizes and within a phylogenetic context.

Some of the initial models of functional responses were based on the feeding of praying mantises (Insecta, Mantodea), which may serve as models to examine allometric relationships of ingestion and compare with other groups. Praying mantises are mesopredators and can serve as bioindicators in their environment, as their presence and abundance have been correlated to species richness and biodiversity (Battiston et al. 2020). Among the 2500+ species, they exhibit a ~40‐fold range in body size, all use their raptorial forelegs for prey capture, and the shearing action of their mandibles to process the live prey outside the body (Brannoch et al. 2017; Oufiero et al. 2016; Oufiero 2020; Svenson and Whiting 2009). Mantises have been documented taking prey much larger than themselves, potentially limited by the grip strength of the forelegs and not by the constraints of mandible morphology (Jehle et al. 1996; Nyffeler et al. 2017). Their prey capture mechanism has been well studied and shown to last milliseconds, making this process relatively negligible to their overall handling time (Copeland and Carlson 1979; Corrette 1990; Maldonado et al. 1967; Oufiero et al. 2016; Oufiero 2022; Oufiero et al. 2024; Prete and Cleal 1996). Their feeding seems to be limited by their satiation, although it can take a large amount of resources to reach that level (Bertsch et al. 2019; Holling 1966; McCue et al. 2016). Furthermore, mantises have evolved some of the most diverse camouflage strategies, including leaves, bark, grass, flowers, and more (Brannoch et al. 2017; De Alcantara Viana et al. 2023; Oufiero 2020; Svenson and Whiting 2009). The evolution of camouflage strategies has recently been shown to result in ecomorphs in other groups and may result in ingestion variation based upon the different micro‐habitats they mimic (Boisseau et al. 2025; Clare et al. 2011; Felice et al. 2019; Law et al. 2018). It has been hypothesized that camouflage evolution can lead to habitat specialization, which may result in different diets among the camouflage strategies (Boratyński et al. 2017). For example, some flower‐mimicking species may be eating more pollinator species, which tend to be soft‐bodied (O'Hanlon et al. 2015; Svenson et al. 2016). Camouflage evolution may therefore select for variation in the time to ingest prey based upon these habitat specializations.

A few studies have examined allometric scaling of ingestion interspecifically among closely related, diverse species and in a phylogenetic context (Coblentz et al. 2025; Vucic‐Pestic et al. 2010). Understanding the interspecific scaling of ingestion would provide information on how this biological rate, which is an important component of handling time, relates to other biological rates such as metabolic rate. It also allows for the determination of species that may adaptively deviate from allometric relations due to variation in ecological factors (Lindstedt and Hoppeler 2023). The goal of this study was to examine the allometric variation in ingestion rate among praying mantis species in a phylogenetic context to test the hypotheses that ingestion rate will scale allometrically and vary with camouflage strategy. Hence, we first determined the repeatability of ingestion rate within individuals among species of praying mantis, as this trait has not been measured before in this or related groups. A repeatable trait ensures the performance metric is capturing a reliable performance metric, is a potential target for natural selection, and can set the upper limits of heritability (Dohm 2002; Lessells and Boag 1987; Oufiero and Garland 2009). Next, we examined the allometric relationship of ingestion rate with body size and determined if camouflage strategies (i.e., potential ecomorphs) vary in their ingestion rate after accounting for body size. We then compared our ingestion times against handling times of other predators (Uiterwaal et al. 2022). We assume our measure of ingestion is a good approximation of a mantis's handling time, as many studies exclude digestion in handling time, and the time to ingest in a mantis can be significantly longer than attacking and subduing. Comparing to other groups in the larger dataset provides information for how this invertebrate mesopredator may impact food web dynamics and if it may have a similar impact to other predatory organisms. Lastly, we relate ingestion scaling to metabolic scaling to determine if they are similar (Chown et al. 2007).

## 2 Materials and Methods

**FIGURE 1:**
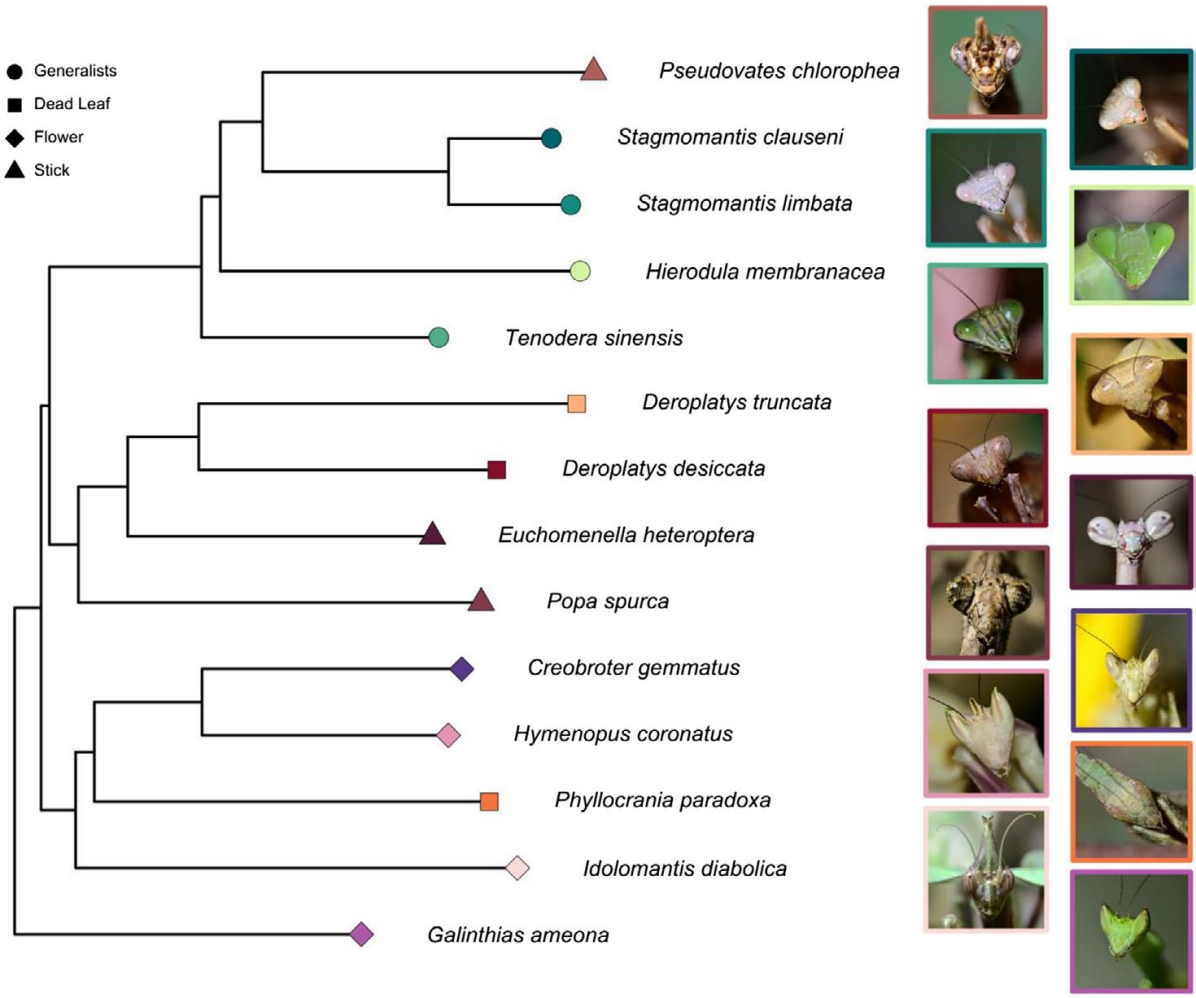
Sampled taxa, evolutionary relationships, and camouflage strategies on a pruned phylogeny derived from Svenson and Whiting (2009). Head shots of each species (not to scale) show the diversity of shapes among sampled taxa. Branch lengths are substitutions per site. All photo credits: M. Wood.

To examine the allometric variation in ingestion, we obtained 1–8 adult female mantises from 14 different species from domestic hobbyists or those that were bred in the lab (Figure 1, Table 1). These species represent 4 different camouflage strategies and multiple taxonomic families: (1) “generalist species” (*Hierodula membranacea, Stagmomantis limbata, Stagmomantis clauseni*, and *Tenodera sinensis*); (2) “flower mimics” (*Creobroter gemmatus, Galianthias ameona, Hymenopus coronatus*, and *Idolomantis diabolica*); (3) “dead leaf mimics” (*Deroplatys desiccata, Deroplatys truncata*, and *Phyllocrania paradoxa*); and (4) “stick mimics” (*Euchomenella heteroptera*, *Popa spurca*, and *Pseudovates chlorophea*). Camouflage was classified by apparent cryptic visual features or lack thereof, similar to other studies (Brannoch et al. 2017; Garikipati et al. 2025; Svenson and Whiting 2009). Generalists were considered to be species that lacked large projections on legs, abdomen, or their pronotum. Mantises came from three sources: (1) bred and nymphs reared to adults in the lab ( *H. coronatus* , *E. heteroptera*, *S. limbata, P* *. paradoxa* ), (2) bred in captivity by private breeders and brought into the lab as nymphs or subadults and reared to adults (*H. membranacea, D. desiccata, D* *. truncata* , *C* *. gemmatus* , *P. spurca*, and *S. clauseni*), or (3) obtained as adults (*I. diabolica* and *T. sinensis* ). Nymphs and subadults were reared to adulthood in 12.7 × 12.7 × 17.8 cm clear containers with fabric gauze on the top and sides for mantis to climb freely. Some of the larger species were kept in 30.48 cm × 30.48 cm × 30.48 cm net cubes (e.g., *H. membranacea* ). All species were fed to satiation twice a week as nymphs up to adults and starved for 72 h prior to filming as adults. Mantises were fed *Drosophila melanogaster* Meigen, 1830 and *Drosophila hydei* Sturtevant, 1921 when young and *Blatta lateralis* Walker, 1868 as older juveniles. All mantises were provided with water every 3 days by a spray bottle. After an individual molted to an adult, its mass was recorded to account for its body size before being fed its next meal. The vivarium room was kept on a 12H:12H day/night cycle, at 24.3°C ± 0.0031 (s.e.m.) and 48.12% ± 0.094 (s.e.m.) relative humidity throughout the study.

**TABLE 1:** Summary of species, sample sizes, and data from the mantises used in this study.

Species	*N*	Predator mass (g)	Prey mass (g)	Prey mass eaten (mm)	Ingestion time (s)	Ingestion rate (mg/s)
*Creobroter gemmatus*	5	0.5567 (0.0504)	0.0456 (0.0075)	0.0449 (0.008)	643.2462 (328.3693)	0.0784 (0.0237)
*Deroplatys desiccata*	4	1.9293 (0.085)	0.0407 (0.0064)	0.0402 (0.007)	292.2632 (100.1567)	0.1538 (0.0677)
*Deroplatys truncata*	3	2.2142 (0.4718)	0.0439 (0.0057)	0.0434 (0.0055)	532.9941 (249.8546)	0.1005 (0.0481)
*Euchomenlla heteroptera*	9	0.6912 (0.2505)	0.0429 (0.0079)	0.0425 (0.0077)	980.1636 (389.2921)	0.0486 (0.0168)
*Galinthias ameona*	3	0.1374 (0.0411)	0.0448 (0.0065)	0.0438 (0.0058)	2211.4154 (495.028)	0.0207 (0.0052)
*Hierodula membranacea*	5	2.4955 (0.1708)	0.0428 (0.0048)	0.0423 (0.0049)	139.2667 (46.0557)	0.3297 (0.0961)
*Hymenopus coronatus*	7	1.0042 (0.2364)	0.0446 (0.0064)	0.0441 (0.0065)	482.6788 (235.1151)	0.1094 (0.05)
*Idolomantis diabolica*	1	3.6555 (0)	0.0514 (0.0117)	0.0512 (0.0116)	134.7 (41.3326)	0.4008 (0.1145)
*Phyllocrania paradoxa*	5	0.6809 (0.0397)	0.0469 (0.0069)	0.0426 (0.0106)	922.5 (323.3614)	0.0523 (0.0232)
*Popa spurca*	6	1.0061 (0.3193)	0.0455 (0.0096)	0.0436 (0.0127)	317.2615 (158.7266)	0.159 (0.0702)
*Pseudovates chlorophea*	3	0.9992 (0.1605)	0.045 (0.0074)	0.0439 (0.0081)	370.675 (154.3986)	0.1325 (0.0425)
*Stagmomantis clauseni*	4	0.4159 (0.0511)	0.0418 (0.0089)	0.0413 (0.0102)	712.2 (211.4387)	0.0643 (0.0257)
*Stagmomantis limbata*	6	0.8661 (0.1579)	0.0462 (0.0092)	0.0459 (0.0093)	463.42 (163.2099)	0.108 (0.0377)
*Tenodera sinensis*	5	3.1597 (0.9497)	0.0406 (0.0077)	0.0405 (0.0076)	136.45 (37.6038)	0.5287 (0.7152)

*Note:* Means (standard deviations) for each species of the traits analyzed are included.

Each mantis (*N* = 66) had its ingestion rate measured 3–5 times for a total of 324 trials. Each mantis was starved for 72 h before feeding trials and recorded using aTLi EON time‐lapse cameras (Atli Time Lapse, Hong Kong) at a rate of 1–2 frames per second. Individuals were removed from their home enclosure and were placed on a platform in front of the time‐lapse camera to acclimate for 1–5 min (Video 1). After the acclimation time, the time‐lapse camera was set to record, and the mantis was presented with a pre‐weighed (to the nearest 10,000th of a gram) live Blue bottle fly ( *Calliphora vomitoria* ) held by tweezers. Once the individual mantis captured the fly, they ingested the prey without being disturbed. The recording was stopped when the mantis stopped eating the prey, even if the whole prey was not ingested. From the videos, the start time (the point at which the mantis grabs the fly) and the stop time (the point when the mantis completed eating) were recorded based on the time stamps provided by the time‐lapse video (Video 1). If the mantis left behind any fragments of the fly, the fragments were weighed to the nearest 10,000th of a gram and subtracted from the full mass of the fly to determine the total mass ingested by the mantis, which was used in subsequent analyses as our measure of prey size. From these data, we determined the ingestion rate as the mass ingested per second by the mantis in each trial by dividing the total mass ingested by the time it took in seconds, resulting in an ingestion rate (mg/s). We also recorded the mantis's mass to the nearest tenth of a milligram, closely following its adult molt to determine overall body size. Lastly, we recorded the date the mantis molted to its adult instar for most mantises and tested all mantises within ~9 months of their age at maturity. The age of some were unknown as *I. diabolica* was obtained as an adult, and *T. sinensis* were obtained from the field as adults. Each filming day, an individual mantis was filmed 1–3 times and capped at 3 flies a day, depending on their willingness to capture the prey and the mantis's size. Smaller species (e.g., *G. ameona*) were only offered 1 fly a day, while larger species (e.g., *I. diabolica*), were offered a maximum of 3 flies a day, which is far less than what has been shown for them to reach satiation, so it ensured mantises were not satiated and their ingestion was not slowed down (Bertsch et al. 2019; Holling 1966).

**VIDEO 1:** Example time‐lapse videos from each camouflage strategy. The time‐lapse videos were recorded at 1–2 frames per second and used to quantify the time it takes mantises to ingest a consistent prey item, Blue bottle flies ( *Calliphora vomitoria* ). Video content can be viewed at https://onlinelibrary.wiley.com/doi/10.1002/ece3.73091.

### 2.1 Statistical Analyses

We log_10_‐transformed the ingestion rate, ingestion time, prey‐specific ingestion time (ingestion time (s)/prey mass ingested (mg)), and predator body mass to normalize the data and match transformations of handling time and metabolic scaling (Chown et al. 2007; Coblentz et al. 2025). Next, we used the ICCest package in R to obtain the intraclass correlation coefficient and the 95% confidence interval (CI) for those estimates to determine the repeatability of log_10_ ingestion rate, time, and prey‐mass‐specific time within individuals (*N* = 66, as each individual was tested 3–5 times total) (Wolak et al. 2012). Repeatability typically ranges from 0 to 1, with low repeatabilities being closer to 0 and higher being closer to 1 (Oufiero and Garland 2009).

To examine the allometric relations between ingestion and predator mass and determine the potential effect of camouflage strategy, we compared three dependent variables related to ingestion time against log_10_ predator mass: (1) log_10_ ingestion rate (mg/s), (2) log_10_ ingestion time (s), and (3) log_10_ prey‐mass‐specific ingestion time (sec./mg). These three dependent variables were chosen to account for differences in predator size and relative predator size to our standard prey item and to compare with other studies (Brose 2010; Chown et al. 2007; Uiterwaal et al. 2022; Vucic‐Pestic et al. 2010). For each dependent variable, we compared models with and without the effect of camouflage and determined the best fit model by their Akaike Information Criterion corrected (AICc) for smaller sample sizes (Burnham and Anderson 2002). This resulted in two models per dependent variable. Lastly, we examined the two models for each dependent variable using a phylogenetic mixed model approach (PGLMM), taking into account within‐species variation and the means of the individuals, and a phylogenetic generalized least‐squares approach (PGLS) on species means. Like other phylogenetic comparative methods (e.g., phylogenetic independent contrasts or PGLS), PGLMMs include the phylogenetic relationships among species as a covariance matrix, but in PGLMMs, the species are included as the random effect, as we used multiple individual means per species (De Villemereuil and Nakagawa 2014b). Since multiple individuals are included per species, as opposed to analyzing species means in PGLS, the within‐species variance can be incorporated to determine if it has any effect on the model. We include both the PGLMM and PGLS to assess the effects of including within‐species variation on our allometric relationships, and due to our lower species‐level variation (*N* = 14 species). This resulted in a total of twelve models, four for each dependent variable (Supporting Information).

We used the MCMCglmm package and function for PGLMM analyses on individual means among species (de Villemereuil and Nakagawa 2014a; Hadfield 2010; Whitlow et al. 2019). The PGLMM models included each mantis species' mean of the continuous independent body mass as well as the variance among individual mantis body mass, calculated as the difference between each mantis species' mean body mass and each mantis's body mass (de Villemereuil and Nakagawa 2014a). A subset of models included the fixed effect of camouflage. Each PGLMM was run 1,000,000 times and included the random effect of the phylogeny and species, with the scale set to true as the phylogeny is not ultrametric (de Villemereuil and Nakagawa 2014a; Whitlow et al. 2019). We used a pruned phylogeny from Svenson and Whiting (2009). If a species was not in the phylogeny, we used another species in the same genus (Garikipati et al. 2025). For example, *Stagmomantis clauseni* is a recently described species, so we used the *Stagmomantis* sp. from the phylogeny instead (Garikipati 2024; Svenson and Whiting 2009). Lastly, we estimated lambda for each model, a measure of phylogenetic signal (De Villemereuil and Nakagawa 2014b). PGLS models were analyzed using the CAPER package for R on species means using the same phylogeny as in the PGLMM analyses (Orme et al. 2013). We used the ANOVA feature in CAPER to assess the significance of the effect of camouflage. We also used the gls.ci function in evomap to obtain CIs around our PGLS regression line (Smaers and Mongle 2014).

To compare the scaling of mantis ingestion to other groups, we used the FoRAGE database that provides fitted handling times for more than 2500 standardized functional responses (Uiterwaal et al. 2022). First, we transformed our ingestion time data into days from seconds to match the handling time in the FoRAGE dataset. We then obtained the relative prey mass‐specific handling time by dividing handling time (in days) by prey mass (in mg) for both our data and the FoRAGE data. While we standardized our prey size, it was not clear if all the studies in the FoRAGE dataset did so. Using prey‐mass‐specific handling time allows us to better compare our results and match those of others (Coblentz et al. 2025). We only use the species means from our dataset to compare. For the FoRAGE dataset, we only included predators and excluded parasitoids. We then split the dataset into major groupings: insects (which includes ~11 families), arachnids, mollusks, reptiles, fishes, and mammals. We chose these groups as there are similar predators within them, as well as invertebrate and vertebrate predators. This allowed us to compare mantises to other insects, as well as other predators. For each group, we aggregate the data to species means. For groups with *N* > 10 species (e.g., insects, arachnids, mammals, fishes), we analyzed the relationship between log_10_ prey‐mass‐specific handling time (mg/days) and log_10_ predator mass using the lm function in R. For the mammal FoRAGE dataset, we excluded one species because of its unusually low prey‐mass‐specific handling time. Because of the diversity of taxa and variation in known phylogenetic relationships, we do not include phylogeny for the data in the FoRAGE dataset. For the mantis data, we include a non‐phylogenetic analysis to compare to the FoRAGE dataset as well as a PGLS analysis (Table 2).

**TABLE 2:** Linear regression models of log_10_ handling time/prey mass on log_10_ predator mass for the mantises in the current dataset and the groups with more than 10 species from the FoRAGE dataset (Uiterwaal et al. 2022).

Group	*N*	*Y*‐intercept	Slope	95% CI slope	*F*	df	Adjusted *R* ^2^	*p*
Mantis: phylogenetic	14	−1.0301	−0.9466	−1.16, −0.728^a^	85.76	1.12	0.8670	< 0.001
Mantis: non‐phylogenetic	14	−1.2842	−0.8676	−1.12, −0.618	57.20	1.12	0.8121	< 0.001
Insects	173	−0.3037	−0.7630	−0.981, −0.545	47.54	1.171	0.2130	< 0.001
Arachnids	63	−0.4306	−1.0624	−1.42, −0.708	35.98	1.61	0.3607	< 0.001
Fish	71	−0.8688	−0.6897	−0.908, −0.472	39.86	1.69	0.3570	< 0.001
Mammals	12	−1.6805	−0.6286	−1.08, −0.179	9.722	1.10	0.4422	0.0109

*Note:* The number of species (*N*) and the results for each model are provided. The mantis dataset was analyzed in a phylogenetic context using phylogenetic generalized least squares (PGLS) and a non‐phylogenetic context to compare with the other groups where a phylogeny was lacking. The regression models provided the best‐fit lines, as shown in Figure 3.

^a^ Confidence intervals (CIs) could not be obtained from pgls.profile because the lambda transformation using maximum likelihood was 1 in this study, suggesting a strong phylogenetic signal in the relationship. However, we were able to obtain them using the following equation: CI = slope + c(−1.1)*standard error slope*qt(0.975.12) in R. df, degrees of freedom.

## 3 Results

### 3.1 Repeatability

All measures of ingestion, log_10_ ingestion rate (ICC = 0.776, 95% CI (0.701, 0.842), *N* = 66), ingestion time (ICC = 0.852, 95% CI (0.797, 0.898), *N* = 66), and prey‐mass‐specific time (ICC = 0.793, 95% CI (0.722, 0.855), *N* = 66) were repeatable among individuals.

### 3.2 Ingestion Rate (mg/s) vs. Predator Mass

**FIGURE 2:**
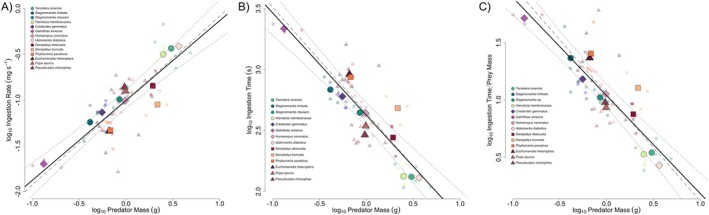
Allometric relationship of (A) log_10_ ingestion rate (mg/s) and log_10_ predator mass (g); (B) log_10_ ingestion time (s) and log_10_ predator mass (g); (C) log_10_ prey‐mass specific time (ingestion time/prey mass) and log_10_ predator mass. Larger points represent species means (used in both phylogenetic mixed models [PGLMM] and phylogenetic generalized least squares [PGLS]), smaller points represent individual means (used in PGLMM). Black line, regression line from PGLMM; dashed gray line, PGLS regression line; dotted gray line, confidence intervals (CIs) for the PGLS regression line. Shapes represent camouflage strategies as depicted in Figure 1, circles = “generalists”, boxes = “dead leaf”, diamond = “flower”, triangle = “stick”. While several species tend to fall outside the CI, they were not significantly different than the other camouflage groups in B and C, but were in A.

For both the PGLMM and PGLS analyses, the models without camouflage were a better fit (PGLMM: with camo AICc = −25.45, without camo = −32.38; PGLS: with camo = −10.92, without camo = −13.23), suggesting that the evolution of camouflage does not significantly influence ingestion rate among mantises (Figure 2A). However, the PGLS model with camouflage had a significant effect of camouflage (*F*_1,3_ = 5.11, *p* = 0.025) with the deadleaf mimics tending to have lower ingestion rates for their body size than the other camouflage groups (Supporting Information, Figure 2A). Since the better fit models were the ones without camouflage, we show the results of those models only.

Ingestion rate scaling was described by the following allometric equations: PGLMM: log_10_ ingestion rate = −1.007 + log_10_ predator mass × 0.875 (*p* < 0.001); PGLS: log_10_ ingestion rate = −1.006 + log_10_ predator mass × 0.923 (*F*_1,12_ = 83.83, adjusted *R* ^2^ = 0.864, *p* < 0.001). In both, there was a significant positive effect of body mass on ingestion rate (Figure 2A). While the PGLMM model had a slightly lower slope, this is likely due to the within‐species variation, which did not have a significant effect (*p* = 0.381). The PGLMM model had a lambda of 0.627, suggesting a relatively strong phylogenetic signal.

### 3.3 Ingestion Time vs. Predator Mass

For the relationship between ingestion time and predator mass, the better fit models were the ones that did not include camouflage strategy (PGLMM: with camo AICc = −104.15, without camo = −111.89; PGLS: with camo = −9.06, without camo = −13.36). For both the PGLMM and PGLS models with camouflage, camouflage did not have a significant effect (*p* > 0.05), although the dead leaf mimics tended to have a higher ingestion time (Figure 2B).

Ingestion time scaling was described by the following allometric equations: PGLMM: log_10_ ingestion time = 2.626 + log_10_ predator mass × −0.841 (*p* < 0.001); PGLS: log_10_ ingestion rate = 2.644 + log_10_ predator mass × −0.869 (*F*_1,12_ = 75.02, Adjusted *R* ^2^ = 0.851, *p* < 0.001). In both, there was a significant negative relationship between ingestion time and predator body mass (Figure 2B). The PGLMM model again had a slightly lower slope, likely due to the inclusion of within‐species variation, which had a marginally significant effect (*ß* = −0.261, *p* = 0.072). The PGLMM model had a lambda of 0.740, again suggesting a relatively strong phylogenetic signal.

### 3.4 Prey‐Mass Specific Ingestion Time vs. Predator Mass

When examining the relationship between prey‐mass‐specific ingestion time and relative predator mass, the models that did not include camouflage were again the better fit (PGLMM: with camo AICc = −23.16, without camo = −29.62; PGLS: with camo = −10.57, without camo = −12.99). In the models with camouflage, the PGLMM and PGLS models did not show a significant relationship of ingestion with camouflage strategies (*p* > 0.05), although the dead leaf mimics tended to have a longer ingestion time in relation to prey mass (Figure 2C).

Prey‐mass‐specific time scaled in relation to relative predator mass based on the following equations: PGLMM: log_10_ prey‐mass‐specific ingestion time = 1.035 + log_10_ predator mass × −0.857 (*p* < 0.001); PGLS: log_10_ prey‐mass‐specific ingestion rate = 1.056 + log_10_ predator mass × −0.942 (*F*_1,12_ = 86.13, adjusted *R* ^2^ = 0.868, *p* < 0.001). In both, there was a significant negative relationship between prey‐mass‐specific ingestion time and predator mass (Figure 2C). The PGLMM model had moderate phylogenetic signal (lambda = 0.607) and no significant effect of within‐species variation (*p* = 0.118).

### 3.5 Prey‐Mass‐Specific Handling Time vs. Predator Mass: Comparison with Other Groups

**FIGURE 3:**
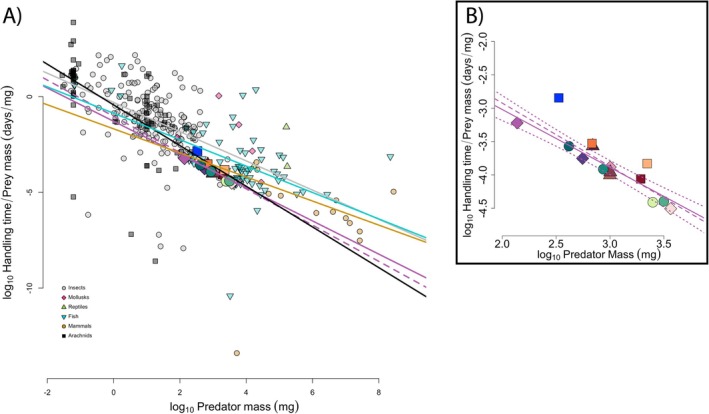
(A) Relationship between log_10_ handling time/prey mass and log_10_ predator mass. Colored and shaped smaller points represent different groups based on the FoRAGE dataset (Uiterwaal et al. 2022). Matching lines represent regression lines for each group with a sample size > 10 species (mollusks and reptiles *N* < 10). (B) Praying mantis data only, including data from this study with fitted regression lines and confidence intervals of the phylogenetic generalized least squares (PGLS) line (dashed purple and dotted purple) and the blue square from the FoRAGE dataset. The FoRAGE data include adults, juveniles, and both sexes. Data from each mantis species collected in this study are the larger points, colored and shaped as in previous figures. The dashed purple line represents a regression line through the mantises based on a PGLS regression using caper. The solid purple line represents a nonphylogenetic regression line to match the other groups from the FoRAGE dataset. The FoRAGE dataset was not corrected for phylogenetic relationships. Blue square represents the only mantis species in the FoRAGE dataset (*Hierodula crassa*). The insect groups (gray circles) include multiple families. The mammal regression line excludes the low outlier, which represents the species *Sorex cinereus cinereus* . FoRAGE data aggregated to species means for each major taxonomic group.

Comparing our measure of ingestion time to handling time of other predators shows that mantises tend to have allometric scaling more comparable to arachnids than to other invertebrates (insects) and vertebrates (mammals and fish) in the FoRAGE dataset (Figure 3). The slope for mantises describing the relationship between prey‐mass‐specific handling time and predator mass (non‐phylogenetic = −0.868, PGLS = −0.947) was close to arachnids (*ß* = −1.062) than to the other insects in the database (*ß* = −0.763), mammals (*ß* = −0.629) or fishes (*ß* = −0.690, Table 2, Figure 3).

## 4 Discussion

We found that (i) the ingestion rate, time, and prey‐mass‐specific time were repeatable among praying mantises. We also found that (ii) a power‐law described the relationship with predator mass with an increasing function for ingestion rate and a decreasing function for ingestion time and prey‐mass‐specific time (Figure 2). Lastly, we found (iii) a minimal effect of camouflage on ingestion, and that mantis ingestion scales similar to the handling time of arachnids FoRAGE dataset (Figure 3) and insects from other studies (Vucic‐Pestic et al. 2010). Together, these results highlight the role of body size in dictating variation in how long it takes praying mantises to ingest a prey, despite their highly variable camouflage strategies. This suggests that their feeding, similar to their prey capture, may allow them to feed on a variety of resources (Garikipati et al. 2025). Comparisons to other groups highlight the similarity in praying mantis feeding to other invertebrate predators (Figure 3), suggesting they might play similar roles in the environment. Praying mantises were some of the earlier organisms used in models of functional responses, and our results will aid their continued use as a model in predator–prey dynamics. With increasing research on their feeding, ecology, and diet, they may serve as further models to understand the evolution of functional responses in relation to body size and ecology.

We found that the rate (ICC = 0.775), time (ICC = 0.852), and prey‐mass‐specific time (ICC = 0.793) to ingest a meal were repeatable within individuals. Repeatability has been examined extensively for other physiological traits and rates, such as locomotion and metabolic rates, but rarely for ingestion (Bell et al. 2009; Nespolo and Franco 2007; Oufiero and Garland 2009). Most studies find consistency within individuals for those traits. For example, the standard metabolic rate (SMR) was found to be repeatable in both male and female cockroaches (*Perisphaeria* sp., *r* = 0.51) (Marais and Chown 2003). If the intake of energy is related to the expenditure of energy and we expect similar allometric scaling, we might expect similarity in the repeatability of these traits. Based on our results, it suggests that the intake of energy among mantises is a repeatable physiological trait. The repeatability of this trait may allow ingestion to act as a target for natural selection and diverge based on varying selective pressures, such as diet, due to microhabitat occupation based on camouflage strategy.

The results for the effects of camouflage were mixed, but suggest that the evolution of camouflage strategies, in this sample, does not influence the time it takes mantises to ingest a prey. While some of the models demonstrated a minimal effect of camouflage, with the dead leaf mimics tending to ingest more slowly, for both the PGLMM and PGLS analyses, models without the fixed effect of camouflage were better, suggesting this trait is not an important predictor of ingestion (Figure 2). This is similar to recent findings on prey capture variation among mantis camouflage strategies, where dead leaf mimics tend to have slower attempts, but other factors, such as phylogeny, also influenced prey capture patterns (Garikipati et al. 2025). The role of camouflage as a selective agent for ecomorphs, where the morphology evolves to match the functioning in the environment, has only recently begun to be explored (Boisseau et al. 2025; Pembury Smith and Ruxton 2020). While flower‐mimicking species, such as the orchid mantis, may encounter more soft‐body pollinating prey than a species feeding in other parts of the environment (O'Hanlon et al. 2013; Svenson et al. 2016), their rate of ingestion does not vary outside of allometric scaling (Figure 2). This suggests that the mandibles used for shearing living prey may be versatile at ingesting different types, allowing mantises to evolve and diversify among different mimicking niches. The slightly reduced ingestion of the dead leaf mimics may be due to variation in the mandible morphology, their behavior, or reduced movement to avoid detection (Khokhar and Soomro 2009; Zeimet et al. 2025). Future studies aimed at quantifying mandible and head variation in relation to diet among camouflage strategies would shed light on their role in ingestion rate variation (Figure 1). For example, does ingestion time vary with prey of different hardnesses, and would a flower mimic that preys on soft‐body pollinators be able to ingest hard prey at the same rate?

We found a power‐law decrease in prey‐mass‐specific handling time of mantises and other groups (Figures 2 and 3), which suggests that the evolution of larger body sizes among predators increases predation rate, which has several advantages. As body size increases, the number and/or quality of offspring also tends to increase; therefore, there may be selection on larger body sizes to increase fecundity (Blanckenhorn 2000). A reduction in time to process a resource and an increase in predation rate may provide the energy needed for an increase in fecundity. The evolution of a larger body size may also increase the resources a predator can obtain. In fact, evidence among flower‐mimicking mantises, including *H. coronatus* , has shown that the evolution of larger females among species is likely due to an increase in predation success and opportunity to acquire pollinators due to their aggressive mimicry, not necessarily fecundity (O'Hanlon et al. 2013, 2015; Svenson et al. 2015, 2016). While *H. coronatus* are not the largest mantis in our dataset, it does suggest that the increased body size also reduces their handling time, which should, in turn, increase predation rate. This would also be beneficial if predation success were related to body size evolution to increase the chances of obtaining additional resources. Whether selection for larger body size is due to fecundity, predation success, or a combination, the reduced handling time should increase energy intake, supporting both.

Interspecific allometric scaling of handling time, taken as the prey‐mass‐specific time, is similar among taxa and studies. For example, our scaling is most similar to arachnids from the FoRAGE dataset, compared to other insects, fish, and mammals, but the CIs for all groups include the other slopes (Figure 3, Table 2). This suggests some commonality in the scaling of the time it takes to ingest energy among different groups. While the results are intriguing, some caution is warranted. First, the handling time from the FoRAGE dataset is extracted from published studies, fitted with a Type II functional response (Uiterwaal et al. 2022). We are assuming our ingestion rate should represent a proxy for this handling time. Comparing our mantis data to the only mantis species in the FoRAGE dataset shows that we obtained faster handling times based on ingestion only. *H. crassa* in the FoRAGE data set is from Holling (1966) and had a mass of 335 mg, which is in the range of our praying mantis masses (100.9–4874 mg), although on the smaller end. However, the prey type was different, and the mass of the prey for *H. crassa* was lower (8.69 mg FoRAGE vs. 43.21 ± 0.047 mg (mean ± s.e.) in our dataset). As a result, this pushes *H. crasssa*'s prey‐mass‐specific handling time off our best fit line and out of our CIs (Figure 3B). While there are many reasons for this discrepancy, we feel our data are a good approximation of the handling time of a mantis when they are limited in obtaining another resource. Second, the handling time from the FoRAGE dataset is based on other studies where lots of potentially confounding variables are not controlled, such as prey size. However, using prey‐mass‐specific handling time helps correct for variation in those relationships (Coblentz et al. 2025). Third, the FoRAGE dataset includes various life stages and sexes, whereas our data is on adult females. We did not limit the FoRAGE data to adult females only, as it would greatly reduce the comparisons, and some of the species' life stages and sex are unknown. However, this variation could also cause some of the discrepancies. Lastly, the relationships for other groups were not phylogenetically corrected due to the diversity of species and the lack of consistent phylogenies for all the species in the FoRAGE dataset. However, comparing the fit of a non‐phylogenetic model to a phylogenetic model in our mantis dataset shows minimal differences between the two, with the non‐phylogenetic relationship within the CIs of the phylogenetic, suggesting that predator mass, rather than phylogeny, better explains the relationships (Figure 3B). If we assume our measure of handling time (i.e., ingestion time) is similar, then it suggests that as predator mass increases, the power‐law decrease in prey‐mass‐specific handling time among mantises is comparable to other groups (Figure 3). This suggests that predator mass may best explain the time it takes a predator to handle a prey. Incorporating additional information on the types of predators and focusing on one life stage and sex might help further our understanding of the role of different types of predators (e.g., sit‐and‐wait vs. active foragers) and their role in the ecosystem.

Handling and ingestion time have been hypothesized to scale similarly to metabolic rate, but in an inverse relationship (Brose 2010; Coblentz et al. 2025; Vucic‐Pestic et al. 2010). That is, as the body size of the predator increases, the time to ingest and handle should decrease. Since ingestion rate is one end of an energy continuum of a consumer, where ingestion is the process of acquiring energy and the metabolic rate is the allocation of that energy to maintenance, growth, and reproduction, it was proposed that the two rates would scale similarly (Coblentz et al. 2025; Yodzis and Innes 1992). We found that prey‐mass‐specific handling time scaled with exponents of −0.868 to −0.947 (non‐phylogenetic and phylogenetic, respectively). This is different from and greater than the scaling of metabolic rate in other insects and mantises, specifically. Chown et al. (2007) found that metabolic rate scaled with an exponent of 0.75 with mass after correcting for phylogeny among 391 species of insects from 16 orders. While the scaling of handling time for our mantises was greater, that of the other insects in the FoRAGE dataset was similar (−0.763). McCue et al. (2016) measured the SMR among four species of mantises within a similar size range as the mantises in our study and found a non‐phylogenetic scaling of 0.745, which they found to not be different from other insects, but again differs from our handling time scaling of mantises.

Comparing the handling time of other groups based upon the FoRAGE dataset to published values of metabolic scaling reveals further discrepancies between the rates (Figure 3). McCue et al. (2016) also estimated a “spider curve” for metabolic rate among 75 species of arachnids, which they found to scale with an exponent of 0.658, which is lower than the “spider curve” of handling time from the FoRAGE dataset (−1.062). Mammals had a lower exponent for handling time than for metabolic scaling (Capellini et al. 2010; Glazier 2005). Metabolic scaling of fishes has been shown to vary widely (Glazier 2005), making comparisons to handling scaling difficult. It is therefore not clear if the rate of energy intake and energy use scales similarly, as has been suggested (Brose 2010; Glazier 2015; Hatton et al. 2019; Lindstedt and Hoppeler 2023; Yodzis and Innes 1992). Some groups tend to have a higher scaling for metabolic rate (e.g., mammals), some tend to have a higher scaling for handling time (e.g., our mantises and arachnids), and some are similar (e.g., insects).

The discrepancy between ingestion and metabolic scaling can be due to several factors, including intra‐ versus inter‐specific scaling, limited studies that measure both, abiotic and biotic factors. For example, Iles (2014) measured the ingestion rate and metabolic rate of six marine invertebrates and found variation in the scaling exponent of both traits within each species. The study demonstrated that temperature had a greater effect on metabolic scaling, but that prey type can affect ingestion scaling, emphasizing the myriad of factors that might affect these allometric relationships. As organisms ingest energy and assimilate it in their bodies, some of that energy is lost to waste (feces and urine). The loss of energy to waste has been assumed not to be affected by consumer size, but it may be a contributing factor to the differences in scaling of handling time and metabolic rate with size (Yodzis and Innes 1992). Morphology may also affect ingestion time without affecting metabolic rate. For instance, fish with pharyngeal jaws have higher handling times than species without (Mcgee et al. 2015), and lizards with smaller heads have higher handling times than those with larger heads (Andrews et al. 1987). Diet has been shown to be a strong selective pressure for feeding morphology and to influence ingestion rate (Felice et al. 2019; Iles 2014; Law et al. 2018). Ingestion rate may be constrained by cranial morphology, whereas the metabolic rate may relate more to digestive morphology, causing the differences in scaling (Vucic‐Pestic et al. 2010). Standardizing prey may not reflect the ingestion rate of a predator that has evolved to ingest another resource, which might cause a difference in metabolic and ingestion scaling. Similarly, many organisms, especially ectotherms, have evolved preferred body temperatures for performance traits, and standardizing temperatures might not reflect the optimal body temperature for ingestion and/or metabolic rate (Angilletta 2009). While there are other factors that could play a role in these differences, more studies that control these factors among species are needed to understand the links among body size, ingestion, and metabolism (Brose 2010). If larger consumers are ingesting prey faster than dictated by the metabolic rate, it suggests that they may have a greater impact on the resources and may push the system's stability to decline (Iles 2014).

Handling time, which includes the time for a predator to ingest a resource, is an important component of a species' functional response, which describes its predation rate and has ecological implications. Our results show among ambush predatory insects a power‐law increase in ingestion rate and a power‐law decrease in ingestion time with predator mass that deviates from metabolic scaling predictions but is similar to arachnids. Incorporating the phylogenetic relationships highlights the role of body size evolution on handling time, which may be related to fecundity selection or predation selection. Together, the results suggest that larger mantis species are consuming resources faster than their metabolic needs, which may impact energy fluxes within the ecosystem. As there are several introduced mantis species around the world, where their ecological impacts are unknown, they may be disrupting the ecosystems. For example, in the continental United States, *Tenodera sinensis* was first introduced more than 100 years ago and is larger than some native species, such as *Stagmomantis carolinensis* so that it may have a greater impact on the ecosystem than the native species. Recent studies using DNA metabarcoding have examined diets of introduced species, which provides more information on the species they are eating (Wilson Rankin et al. 2023). More diet information could be useful to understand the size and type of prey selected and how it relates to their ingestion and metabolic rates. Integrating diet, morphology, and measures of energy intake and use among species in a phylogenetic context will provide more detailed information on the evolution of functional responses, which may be useful to understand how predators, including introduced species, impact the ecosystem.

## Author Contributions

**Christopher Oufiero:** conceptualization (lead), formal analysis (lead), investigation (supporting), methodology (supporting), project administration (lead), resources (lead), supervision (lead), validation (lead), writing – original draft (lead). **Marlena Wood:** conceptualization (equal), data curation (equal), investigation (equal), methodology (equal), writing – review and editing (equal). **Elizabeth McMillan:** data curation (equal), investigation (equal), methodology (equal), writing – review and editing (equal).

## Conflicts of Interest

The authors declare no conflicts of interest.

## Supporting information


**Data S1:** ece373091‐sup‐0001‐DataS1.zip.

## Data Availability

The raw data are provided as a .csv file with an R Markdown script and output. The R Markdown output has all analyses, results, and figures, including supplementary results not included in the main text of the manuscript. The phylogeny used is from Svenson and Whiting (2009) and can be obtained from that publication.
