# Supplementary material for: Allometry of Ingestion Among Habitat Mimicking Praying Mantises
Source: Ecol Evol. 2026 Feb 13;16(2):e73091. doi: 10.1002/ece3.73091 (PMC12998259; doi:10.1002/ece3.73091)
Supplement: Supplementary file 2 — Data S1: ece373091‐sup‐0001‐DataS1.zip. [file ECE3-16-e73091-s001.zip › Mantis-ingestion.docx]

Mantis ingestion

XXX

2025-08-13

Table of Contents

# Overview

This R Markdown provides the analyses for XX manuscript investigating the allometric relationship among praying mantis species. It also compares mantis allometric relationships to the FoRAGE dataset (<https://knb.ecoinformatics.org/view/doi%3A10.5063%2FF1RX99KB>)

## Read in libraries

library(phytools)

## Loading required package: ape

## Loading required package: maps

library(geiger)
library(MCMCglmm)

## Loading required package: Matrix

## Loading required package: coda

library(emmeans)
library(caper)

## Loading required package: MASS

## Loading required package: mvtnorm

library(MuMIn)

## Registered S3 method overwritten by 'MuMIn':
## method from
## nobs.pgls caper

library(ICC)

## Read in data files and transform variables

tree<-read.nexus("Mantis.nex.txt")#Svenson and Whiting 2009 phylogeny
data<-read.csv("Ingestion rate.csv",stringsAsFactors=TRUE)#mantis data
data$IG.mg<-data$mass.second..g.*1000#set ingestion rate to mg/s
data$lg.IG<-log10(data$IG.mg)
data$lg.time<-log10(data$Real.time..s.)
data$lg.prey.pred<-log10(data$mass.whole.prey.mass.of.mantis)#relative prey size
data$lg.mass<-log10(data$Adult.mass..viv.log.)


#convert ingestion time from seconds to days to match FoRAGE dataset
data$time.h<-data$Real.time..s./60/60/24
data$Mass.mg<-data$Adult.mass..viv.log.*1000#predator mass in mg
data$Mass.prey.mg<-data$Total.Mass.Ingested..g.*1000

#To match more recent allometric relationships Coblentz EA 2025 h/prey mass proportional to predator mass
data$H.prey.mass.s<-data$Real.time..s./data$Mass.prey.mg#sec/mg
data$H.prey.mass<-data$time.h/data$Mass.prey.mg#hours/mg
data$pred.prey<-data$Mass.mg/data$Mass.prey.mg
data$lg.pred.prey<-log10(data$pred.prey)

data.for<-read.csv("FoRAGE_db_V5_Dec_20_2024_sources_and_meta.csv", stringsAsFactors=TRUE)#newer version

data.for$IG<-data.for$Prey.mass..mg./data.for$Fittted.h..day.
data.for$H.prey.mass<-data.for$Fittted.h..day./data.for$Prey.mass..mg.
data.for$pred.prey<-data.for$Predator.mass..mg./data.for$Prey.mass..mg.
data.for$lg.pred.prey<-log10(data.for$pred.prey)
data.for$lg.pred.mass<-log10(data.for$Predator.mass..mg.)
data.for$lg.h<-log10(data.for$Fittted.h..day.)
#subset for invertebrate predator
newdata<-subset(data.for[c(data.for[,3]!="Parasitoid"),])

ph<-c(31,34,43,61,62,63,64,65)#new dataset columns pulling out predator mass, prey mass h in days, pre.prey, lg pred.prey, lg.pred.mass
Mol.data<-subset(newdata[c(newdata[,10]=="Mollusk"),])
Mol.data<-aggregate(Mol.data[,ph], by=list(Mol.data$Predator.scientific.name),
FUN=mean, na.rm=TRUE)
Rep.data<-subset(newdata[c(newdata[,10]=="Reptile"),])
Rep.data<-aggregate(Rep.data[,ph], by=list(Rep.data$Predator.scientific.name),
FUN=mean, na.rm=TRUE)
Arach.data<-subset(newdata[c(newdata[,10]=="Arachnid"),])
Arach.data<-aggregate(Arach.data[,ph], by=list(Arach.data$Predator.scientific.name),
FUN=mean, na.rm=TRUE)
Mamm.data<-subset(newdata[c(newdata[,10]=="Mammal"),])
Mamm.data<-aggregate(Mamm.data[,ph], by=list(Mamm.data$Predator.scientific.name),
FUN=mean, na.rm=TRUE)
Fish.data<-subset(newdata[c(newdata[,10]=="Fish"),])
Fish.data<-aggregate(Fish.data[,ph], by=list(Fish.data$Predator.scientific.name),
FUN=mean, na.rm=TRUE)


newdata<-subset(newdata[c(newdata[,9]=="Invertebrate"),])
newdata<-subset(newdata[c(newdata[,10]=="Insect"),])
newdata<-aggregate(newdata[,ph], by=list(newdata$Predator.scientific.name),
FUN=mean, na.rm=TRUE)

tr<-c(23,24,25,26,13,22,18,27,28,29,30,31,32,33)
data.1<-aggregate(data[,tr], by=list(Tip.label=data[,2],data$Scientific.Name),
FUN=mean, na.rm=TRUE)

colnames(data.1)[2] ="species"
#prunning the tree to match morphology data
row.names(data.1)<-data.1[,1]
foo<-name.check(tree, data.1)
print(foo)

## $tree_not_data
## [1] "Acanthops_falcataria_MN112"
## [2] "Acanthops_sp._MN085"
## [3] "Acontista_sp._MN111"
## [4] "Acromantis_insularis_MN075"
## [5] "Acromantis_montana_MN082"
## [6] "Acromantis_sp._MN004"
## [7] "Acromantis_sp._MN321"
## [8] "Acromantis_sp._MN339"
## [9] "Aethalochroa_sp._MN264"
## [10] "Amantis_biroi_MN083"
## [11] "Amantis_reticulata_MN003"
## [12] "Amantis_sp._MN209"
## [13] "Amantis_tristis_MN333"
## [14] "Amorphoscelis_annulicornis_MN061"
## [15] "Amorphoscelis_austrogermanica_MN176"
## [16] "Amorphoscelis_borneana_MN103"
## [17] "Amorphoscelis_borneana_MN318"
## [18] "Amorphoscelis_singaporana_MN081"
## [19] "Amorphoscelis_sp._MN240"
## [20] "Amorphoscelis_sp._MN317"
## [21] "Amorphoscelis_sp._MN327"
## [22] "Amorphoscelis_sp._MN337"
## [23] "Anasigerpes_bifasciata_MN241"
## [24] "Anaxarcha_intermedia_MN253"
## [25] "Anaxarcha_limbata_MN080"
## [26] "Antemna_rapax_MN147"
## [27] "Antistia_maculipennis_MN305"
## [28] "Archimantis_sobrina_MN012"
## [29] "Austrovates_variegata_MN084"
## [30] "Bantia_werneri_MN115"
## [31] "Bimantis_malaccana_MN331"
## [32] "Blatta_orientalis_BL097"
## [33] "Blattella_germanica_BL082"
## [34] "Blepharopsis_mendica_MN270"
## [35] "Bolbe_pallida_MN021"
## [36] "Bolbe_pygmea_MN040"
## [37] "Bolbella_punctigera_MN189"
## [38] "Bolbena_hottentotta_MN051"
## [39] "Bolbena_hottentotta_MN258"
## [40] "Bolbena_maraisi_MN043"
## [41] "Bolbena_sp._MN292"
## [42] "Brunneria_sp._MN124"
## [43] "Calofulcinia_sp._MN251"
## [44] "Camelomantis_moultoni_MN130"
## [45] "Cardioptera_squalodon_MN178"
## [46] "Carrikerella_ceratophora_MN148"
## [47] "Caudatoscelis_marmorata_MN239"
## [48] "Ceratocrania_macra_MN328"
## [49] "Ceratomantis_ghatei_MN204"
## [50] "Ceratomantis_kimberlae_MN326"
## [51] "Chaeteessa_valida_MN217"
## [52] "Chloroharpax_modesta_MN234"
## [53] "Choeradodis_rhombicollis_MN016"
## [54] "Choeradodis_stalii_MN127"
## [55] "Chroicoptera_saussurei_MN182"
## [56] "Chrysomantis_cachani_MN225"
## [57] "Chrysomantis_sp._MN005"
## [58] "Cilnia_humeralis_MN308"
## [59] "Citharomantis_falcata_MN102"
## [60] "Ciulfina_biseriata_MN036"
## [61] "Cliomantis_cornuta_MN055"
## [62] "Cliomantis_obscura_MN054"
## [63] "Compsothespis_sp._MN282"
## [64] "Congoharpax_aberrans_MN229"
## [65] "Coptopteryx_sp._MN059"
## [66] "Coptopteryx_sp._MN136"
## [67] "Coptotermes_lacteus_IS041"
## [68] "Creobroter_laevicollis_MN073"
## [69] "Creobroter_sp._MN092"
## [70] "Cryptocercus_kyebangensis_BL115"
## [71] "Cryptocercus_russie_BL119"
## [72] "Cryptocercus_scioto_BL120"
## [73] "Cryptotermes_brevis_IS021"
## [74] "Dactylopteryx_flexuosa_MN242"
## [75] "Danuria_thunbergi_MN277"
## [76] "Danuriini_sp._MN032"
## [77] "Danuriini_sp._MN307"
## [78] "Deiphobe_sp._MN256"
## [79] "Deiphobe_sp._MN265"
## [80] "Deiphobella_laticeps_MN066"
## [81] "Deroplatys_rhombica_MN272"
## [82] "Deroplatys_rhombica_MN325"
## [83] "Deroplatys_rhombica_MN347"
## [84] "Deroplatys_sp._MN168"
## [85] "Didymocorypha_lanceolata_MN087"
## [86] "Dysaules_himalayanus_MN088"
## [87] "Dysaules_longicollis_MN202"
## [88] "Dysaules_sp._MN067"
## [89] "Dystacta_alticeps_MN187"
## [90] "Dystacta_alticeps_MN188"
## [91] "Dystactula_grisea_MN197"
## [92] "Elmantis_nira_MN254"
## [93] "Elmantis_nira_MN296"
## [94] "Elmantis_trincomaliae_MN071"
## [95] "Elmantis_trincomaliae_MN261"
## [96] "Empusa_guttula_MN132"
## [97] "Empusa_sp._MN131"
## [98] "Entella_delalandi_MN183"
## [99] "Entella_orientalis_MN304"
## [100] "Entelloptera_rogenhoferi_MN300"
## [101] "Eomantis_guttatipennis_MN074"
## [102] "Eomantis_iridipennis_MN208"
## [103] "Ephestiasula_intermedia_MN063"
## [104] "Ephippiomantis_ophirensis_MN330"
## [105] "Episcopomantis_chalybea_MN034"
## [106] "Eremiaphila_rotundipennis_MN064"
## [107] "Eremiaphila_sp._MN192"
## [108] "Eremoplana_iufelix_MN159"
## [109] "Euantissa_pulchra_MN077"
## [110] "Euantissa_pulchra_MN201"
## [111] "Eumusonia_livida_MN123"
## [112] "Galepsus_ulricae_MN259"
## [113] "Galinthias_amoena_MN266"
## [114] "Gildella_suavis_MN098"
## [115] "Gimantis_insularis_MN100"
## [116] "Gimantis_insularis_MN332"
## [117] "Gongylus_gongylodes_MN006"
## [118] "Gonypeta_borneana_MN097"
## [119] "Gonypeta_borneana_MN324"
## [120] "Gonypetella_sp._MN293"
## [121] "Gonypetyllis_semuncialis_MN089"
## [122] "Gonypetyllis_semuncialis_MN210"
## [123] "Gyna_capucina_BL048"
## [124] "Gyromantis_occidentalis_MN053"
## [125] "Gyromantis_occidentalis_MN056"
## [126] "Haania_lobiceps_MN315"
## [127] "Hagiomantis_superba_MN215"
## [128] "Hapalomantis_congica_katangica_MN291"
## [129] "Hapalomantis_orba_MN280"
## [130] "Hapalopeza_nilgirica_MN070"
## [131] "Hapalopeza_nitens_MN316"
## [132] "Hestiasula_inermis_MN199"
## [133] "Hestiasula_masoni_MN297"
## [134] "Hestiasula_phyllopus_MN340"
## [135] "Heterochaeta_strachani_MN160"
## [136] "Heterochaetula_fissispinis_MN207"
## [137] "Hierodula_schultzei_MN044"
## [138] "Hierodula_sp._MN019"
## [139] "Hierodula_sp._MN343"
## [140] "Hierodulella_celebensis_MN345"
## [141] "Hierodulella_reticulata_MN342"
## [142] "Hodotermes_mossambicus_IS014"
## [143] "Hoplocorypha_sp._MN035"
## [144] "Hoplocorypha_sp._MN042"
## [145] "Hoplocorypha_sp._MN139"
## [146] "Hoplocorypha_sp._MN190"
## [147] "Humbertiella_ocularis_MN093"
## [148] "Humbertiella_ocularis_MN334"
## [149] "Humbertiella_similis_MN069"
## [150] "Idolomorpha_dentifrons_MN175"
## [151] "Indomenella_indica_MN079"
## [152] "Iris_oratoria_MN194"
## [153] "Junodia_amoena_MN286"
## [154] "Kalotermes_flavicollis_IS027"
## [155] "Kongobatha_diademata_MN306"
## [156] "Leptocola_stanleyana_MN284"
## [157] "Leptomantella_albella_MN095"
## [158] "Leptomantella_albella_MN319"
## [159] "Leptomantella_sp._MN108"
## [160] "Leptomantella_sp._MN198"
## [161] "Leptomantella_sp._MN322"
## [162] "Ligaria_brevicollis_ignota_MN138"
## [163] "Ligaria_brevicollis_ignota_MN211"
## [164] "Ligaria_brevicollis_ignota_MN303"
## [165] "Ligariella_gracilis_MN041"
## [166] "Ligariella_trigonalis_MN260"
## [167] "Ligentella_beieri_MN299"
## [168] "Ligentella_zairensis_MN213"
## [169] "Litaneutria_minor_MN045"
## [170] "Liturgusa_maya_MN145"
## [171] "Liturgusa_sp._MN116"
## [172] "Macromantis_nicaraguae_MN144"
## [173] "Macromusonia_conspersa_MN126"
## [174] "Macrotermes_subhyalinus_IS095"
## [175] "Mantis_religiosa_MN001"
## [176] "Mantis_religiosa_MN247"
## [177] "Mantoida_schraderi_MN009"
## [178] "Mantoida_sp._MN109"
## [179] "Mantoida_sp._MN110"
## [180] "Mantoida_sp._MN179"
## [181] "Mantoida_sp._MN180"
## [182] "Mastotermes_darwiniensis_IS034"
## [183] "Metallyticus_fallax_MN157A"
## [184] "Metallyticus_fallax_MN157B"
## [185] "Metallyticus_splendidus_MN156A"
## [186] "Metallyticus_splendidus_MN156B"
## [187] "Metilia_boliviana_MN311"
## [188] "Metilia_brunnerii_MN146"
## [189] "Microphotina_vitripennis_MN271"
## [190] "Miomantis_aurea_MN228"
## [191] "Miomantis_paykullii_MN245"
## [192] "Miomantis_sp._MN181"
## [193] "Miomantis_sp._MN191"
## [194] "Miomantis_sp._MN196"
## [195] "Miromantis_mirandula_MN323"
## [196] "Miromantis_mirandula_MN338"
## [197] "Musoniella_sp._MN122"
## [198] "Musoniella_sp._MN137"
## [199] "Myrcinus_tuberosus_MN257"
## [200] "Namamantis_nigropunctata_MN302"
## [201] "Neomantis_hyalina_MN052"
## [202] "Nilomantis_edmundsi_MN244"
## [203] "Nothogalepsus_planivertex_MN186"
## [204] "Nothogalepsus_sp._MN174"
## [205] "Oligonicella_punctulata_MN049"
## [206] "Oligonicella_scudderi_MN057"
## [207] "Omomantis_zebrata_MN276"
## [208] "Oromantis_sp._MN152"
## [209] "Orthodera_novaezealandiae_MN007"
## [210] "Orthodera_sp._MN033"
## [211] "Orthoderella_ornata_MN060"
## [212] "Orthoderella_ornata_MN129"
## [213] "Otomantis_rendalli_MN290"
## [214] "Otomantis_scutigera_MN155"
## [215] "Otomantis_sp._MN161"
## [216] "Oxyophthalma_engaea_MN200"
## [217] "Oxyopsis_sp._MN294"
## [218] "Oxyothespis_sp._MN283"
## [219] "Oxypiloidea_subcornuta_MN281"
## [220] "Oxypiloidea_tridens_MN289"
## [221] "Oxypilus_hamatus_MN246"
## [222] "Oxypilus_masutus_MN205"
## [223] "Oxypilus_nigericus_MN287"
## [224] "Oxypilus_sp._MN154"
## [225] "Oxypilus_transvalensis_MN172"
## [226] "Panurgica_compressicollis_MN232"
## [227] "Panurgica_fratercula_MN227"
## [228] "Paragalepsus_toganus_MN243"
## [229] "Paraoxypilus_tasmaniensis_MN022"
## [230] "Paraoxypilus_verreauxii_MN008"
## [231] "Parasphendale_sp._MN195"
## [232] "Parastagmatoptera_sp._MN028"
## [233] "Parastagmatoptera_sp._MN125"
## [234] "Parastagmatoptera_sp._MN151"
## [235] "Parathespis_humbertiana_MN263"
## [236] "Photina_sp._MN295"
## [237] "Phyllothelys_decipiens_MN101"
## [238] "Phyllothelys_decipiens_MN329"
## [239] "Phyllothelys_westwoodi_MN076"
## [240] "Phyllovates_cingulata_MN214"
## [241] "Plistospilota_guineensis_MN236"
## [242] "Polyspilota_aeruginosa_MN167"
## [243] "Polyspilota_aeruginosa_MN248"
## [244] "Popa_undata_MN164"
## [245] "Prohierodula_ornatipennis_MN249"
## [246] "Pseudocreobotra_occellata_MN017"
## [247] "Pseudocreobotra_occellata_MN231"
## [248] "Pseudoharpax_uganda_MN170"
## [249] "Pseudomantis_albofimbriata_MN314"
## [250] "Pseudomiopteryx_guyanensis_MN114"
## [251] "Pseudothespis_meghalayensis_MN206"
## [252] "Pseudovates_denticulata_MN312"
## [253] "Psychomantis_borneensis_MN320"
## [254] "Pyrgomantis_jonesi_MN224"
## [255] "Pyrgomantis_nasuta_MN184"
## [256] "Raptrix_fusca_MN113"
## [257] "Raptrix_persiara_MN203"
## [258] "Raptrix_perspicua_MN177"
## [259] "Reticulitermes_santonensis_IS054"
## [260] "Rhomantis_moultoni_MN106"
## [261] "Rhombodera_basalis_MN344"
## [262] "Rogermantis_royi_MN301"
## [263] "Sceptuchus_simplex_MN104"
## [264] "Schizocephala_bicornis_MN065"
## [265] "Sibylla_dives_MN285"
## [266] "Sibylla_operosa_MN226"
## [267] "Sibylla_pretiosa_MN135"
## [268] "Sibylla_pretiosa_MN173"
## [269] "Sphodromantis_lineola_MN015"
## [270] "Sphodromantis_viridis_MN013"
## [271] "Sphodropoda_moesta_MN275"
## [272] "Stagmatoptera_sp._MN029"
## [273] "Stagmatoptera_sp._MN117"
## [274] "Stagmomantis_carolina_MN023"
## [275] "Stagmomantis_sp._MN149"
## [276] "Stagmomantis_sp._MN150"
## [277] "Stagmomantis_sp._MN278"
## [278] "Stagmomantis_sp._MN279"
## [279] "Stagmomantis_vicina_MN024"
## [280] "Statilia_apicalis_MN048"
## [281] "Statilia_apicalis_MN050"
## [282] "Statilia_maculata_MN062"
## [283] "Statilia_maculata_MN336"
## [284] "Statilia_maculata_MN346"
## [285] "Statilia_nemoralis_MN078"
## [286] "Statilia_sp._MN255"
## [287] "Stenomantis_novaeguineae_MN030"
## [288] "Stenomantis_novaeguineae_MN037"
## [289] "Stenomantis_novaeguineae_MN038"
## [290] "Stenomantis_novaeguineae_MN039"
## [291] "Stenopyga_ziela_MN235"
## [292] "Stenotoxodera_porioni_MN090"
## [293] "Supella_longipalpa_BL138"
## [294] "Tamolanica_tamolana_MN020"
## [295] "Tarachina_occidentalis_MN237"
## [296] "Tarachina_sp._MN140"
## [297] "Tarachodes_afzelii_MN158"
## [298] "Tarachodes_afzelii_MN238"
## [299] "Tarachodes_dissimulator_MN233"
## [300] "Tarachodes_sp._MN134"
## [301] "Tarachodula_pantherina_MN309"
## [302] "Tarachomantis_caldwellii_MN310"
## [303] "Taumantis_ehrmannii_MN163"
## [304] "Tenodera_costalis_MN025"
## [305] "Termes_hispaniolae_IS191"
## [306] "Theopompella_chopardi_MN230"
## [307] "Theopropus_elegans_MN094"
## [308] "Theopropus_elegans_MN166"
## [309] "Thesprotia_graminis_MN058"
## [310] "Thesprotia_macilenta_MN313"
## [311] "Thesprotia_sp._MN121"
## [312] "Thesprotiella_peruana_MN269"
## [313] "Thesprotiella_sp._MN252"
## [314] "Thrinaconyx_fumosa_MN046"
## [315] "Thrinaconyx_fumosa_MN086"
## [316] "Thrinaconyx_kirschianus_MN216"
## [317] "Toxoderopsis_taurus_MN068"
## [318] "Tropidomantis_tenera_MN096"
## [319] "Tropidomantis_tenera_MN107"
## [320] "Tropidomantis_tenera_MN212"
## [321] "Tropidomantis_tenera_MN341"
## [322] "Tylomantis_sp._MN047"
## [323] "Vates_pectinacornis_MN014"
## [324] "Vates_sp._MN118"
## [325] "Vates_sp._MN119"
## [326] "Vates_sp._MN128"
## [327] "Xanthomantis_mantispoides_MN099"
## [328] "Xanthomantis_mantispoides_MN335"
## [329] "Xystropeltis_sp._MN268"
## [330] "Yersinia_mexicana_MN273"
##
## $data_not_tree
## character(0)

#remove tips from tree with no data
tree.1<-drop.tip(tree, foo$tree_not_data)
plot(tree.1)


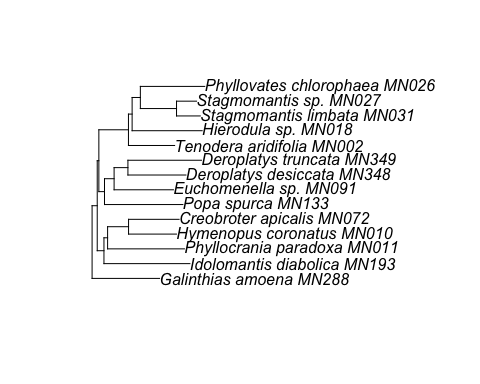


overlap <- name.check(tree.1, data=data.1)
overlap

## [1] "OK"

is_tip <- tree.1$edge[,2] <= length(tree.1$tip.label)
ordered_tips <- tree.1$edge[is_tip, 2]
tree.1$tip.label[ordered_tips]

## [1] "Galinthias_amoena_MN288" "Idolomantis_diabolica_MN193"
## [3] "Phyllocrania_paradoxa_MN011" "Hymenopus_coronatus_MN010"
## [5] "Creobroter_apicalis_MN072" "Popa_spurca_MN133"
## [7] "Euchomenella_sp._MN091" "Deroplatys_desiccata_MN348"
## [9] "Deroplatys_truncata_MN349" "Tenodera_aridifolia_MN002"
## [11] "Hierodula_sp._MN018" "Stagmomantis_limbata_MN031"
## [13] "Stagmomantis_sp._MN027" "Phyllovates_chlorophaea_MN026"

data.1<-data.1[tree.1$tip.label,]
attach(data.1)

foo<-name.check(tree.1, data.1)
print(foo)

## [1] "OK"

# Figure 1, Phylogeny plot

###Figure 1. Phylogeny of species used
#adding in for species means
dcol<-hcl.colors(6, palette="OrRd")
fcol<-hcl.colors(4, palette="PurpOr")
scol<-hcl.colors(6, palette="BrwnYl")
gcol<-hcl.colors(6, palette="Emrld")
len<-nrow(data.1)
for (i in 1:len){
 if(data.1$species[i]=="Creobroter gemmatus"){data.1$col[i]<-fcol[1]}
 if(data.1$species[i]=="Deroplatys dessicata"){data.1$col[i]<-dcol[1]}
 if(data.1$species[i]=="Deroplatys truncata"){data.1$col[i]<-dcol[4]}
 if(data.1$species[i]=="Euchomenlla heteroptera"){data.1$col[i]<-scol[1]}
 if(data.1$species[i]=="Galinthias ameona"){data.1$col[i]<-fcol[2]}
 if(data.1$species[i]=="Hierodula membranacea"){data.1$col[i]<-gcol[6]}
 if(data.1$species[i]=="Hymenopus coronatus"){data.1$col[i]<-fcol[3]}
 if(data.1$species[i]=="Idolomantis diabolica" ){data.1$col[i]<-fcol[4]}
 if(data.1$species[i]=="Phyllocrania paradoxa"){data.1$col[i]<-dcol[3]}
 if(data.1$species[i]=="Popa spurca"){data.1$col[i]<-scol[2]}
 if(data.1$species[i]=="Pseudovates chlorophea"){data.1$col[i]<-scol[3]}
 if(data.1$species[i]=="Sonora Tiger"){data.1$col[i]<-gcol[2]}
 if(data.1$species[i]=="Stagomantis limbata"){data.1$col[i]<-gcol[3]}
 if(data.1$species[i]=="Tenodera sinensis"){data.1$col[i]<-gcol[4]}
 }

len<-nrow(data.1)
for (i in 1:len){
 if(data.1$species[i]=="Creobroter gemmatus"){data.1$sh[i]<-23}
 if(data.1$species[i]=="Deroplatys dessicata"){data.1$sh[i]<-22}
 if(data.1$species[i]=="Deroplatys truncata"){data.1$sh[i]<-22}
 if(data.1$species[i]=="Euchomenlla heteroptera"){data.1$sh[i]<-24}
 if(data.1$species[i]=="Galinthias ameona"){data.1$sh[i]<-23}
 if(data.1$species[i]=="Hierodula membranacea"){data.1$sh[i]<-21}
 if(data.1$species[i]=="Hymenopus coronatus"){data.1$sh[i]<-23}
 if(data.1$species[i]=="Idolomantis diabolica" ){data.1$sh[i]<-23}
 if(data.1$species[i]=="Phyllocrania paradoxa"){data.1$sh[i]<-22}
 if(data.1$species[i]=="Popa spurca"){data.1$sh[i]<-24}
 if(data.1$species[i]=="Pseudovates chlorophea"){data.1$sh[i]<-24}
 if(data.1$species[i]=="Sonora Tiger"){data.1$sh[i]<-21}
 if(data.1$species[i]=="Stagomantis limbata"){data.1$sh[i]<-21}
 if(data.1$species[i]=="Tenodera sinensis"){data.1$sh[i]<-21}
 }

for (i in 1:len){
 if(data.1$species[i]=="Creobroter gemmatus"){data.1$Ecol[i]<-"flower"}
 if(data.1$species[i]=="Deroplatys dessicata"){data.1$Ecol[i]<-"dead leaf"}
 if(data.1$species[i]=="Deroplatys truncata"){data.1$Ecol[i]<-"dead leaf"}
 if(data.1$species[i]=="Euchomenella heteroptera"){data.1$Ecol[i]<-"stick"}
 if(data.1$species[i]=="Galinthias ameona"){data.1$Ecol[i]<-"flower"}
 if(data.1$species[i]=="Hierodula membranacea"){data.1$Ecol[i]<-"generalist"}
 if(data.1$species[i]=="Hymenopus coronatus"){data.1$Ecol[i]<-"flower"}
 if(data.1$species[i]=="Idolomantis diabolica" ){data.1$Ecol[i]<-"flower"}
 if(data.1$species[i]=="Phyllocrania paradoxa"){data.1$Ecol[i]<-"dead leaf"}
 if(data.1$species[i]=="Popa spurca"){data.1$Ecol[i]<-"stick"}
 if(data.1$species[i]=="Pseudovates chlorophea"){data.1$Ecol[i]<-"stick"}
 if(data.1$species[i]=="Sonora Tiger"){data.1$Ecol[i]<-"generalist"}
 if(data.1$species[i]=="Stagomantis limbata"){data.1$Ecol[i]<-"generalist"}
 if(data.1$species[i]=="Tenodera sinensis"){data.1$Ecol[i]<-"generalist"}
 }
tree.2<-tree.1
data.3<-data.1
data.3$species <- trimws(data.3$species) #trimws removes leading or trailing character space
#ok this works
test_sp_df <- as.factor(data.3$species)
droplevels(test_sp_df, exclude = "")

## [1] Creobroter gemmatus Deroplatys dessicata Deroplatys truncata
## [4] Euchomenlla heteroptera Galinthias ameona Hierodula membranacea
## [7] Hymenopus coronatus Idolomantis diabolica Phyllocrania paradoxa
## [10] Pseudovates chlorophea Popa spurca Stagomantis limbata
## [13] Sonora Tiger Tenodera sinensis
## 14 Levels: Creobroter gemmatus Deroplatys dessicata ... Tenodera sinensis

data.3$species <- test_sp_df

is_tip <- tree.2$edge[,2] <= length(tree.2$tip.label)
ordered_tips <- tree.2$edge[is_tip, 2]
tree.2$tip.label[ordered_tips]

## [1] "Galinthias_amoena_MN288" "Idolomantis_diabolica_MN193"
## [3] "Phyllocrania_paradoxa_MN011" "Hymenopus_coronatus_MN010"
## [5] "Creobroter_apicalis_MN072" "Popa_spurca_MN133"
## [7] "Euchomenella_sp._MN091" "Deroplatys_desiccata_MN348"
## [9] "Deroplatys_truncata_MN349" "Tenodera_aridifolia_MN002"
## [11] "Hierodula_sp._MN018" "Stagmomantis_limbata_MN031"
## [13] "Stagmomantis_sp._MN027" "Phyllovates_chlorophaea_MN026"

#data.1<-data.1[tree.1$tip.label[ordered_tips],]
#matching tree to data
data.3<-data.3[tree.2$tip.label,]
attach(data.3)

## The following objects are masked from data.1:
##
## Adult.mass..viv.log., H.prey.mass, H.prey.mass.s, IG.mg, lg.IG,
## lg.mass, lg.pred.prey, lg.prey.pred, lg.time, Mass.mg,
## Mass.prey.mg, pred.prey, Real.time..s., species, time.h, Tip.label

name.check(tree.2, data.3)

## [1] "OK"

new_tiplabels <- c("Creobroter gemmatus", "Deroplatys desiccata","Deroplatys truncata", "Euchomenella heteroptera","Galinthias ameona","Hierodula membranacea", "Hymenopus coronatus", "Idolomantis diabolica","Phyllocrania paradoxa","Pseudovates chlorophea", "Popa spurca ", "Stagmomantis limbata", "Stagmomantis clauseni", "Tenodera sinensis")
tree.2$tip.label<-new_tiplabels

#png(file="Mantis ingestion phylogeny 2.png", height=16, width=16, units="in",res=300)

par(mar=c(5.1,8,4.1,2.1))
plot(tree.2,edge.width=4,align.tip.label=FALSE,label.offset=0.01,cex=2, tip.color="black")
#axisPhylo()
tiplabels(tip=c(1:length(tree.1$tip.label)), pch=data.1$sh, bg=data.1$col,cex=4)
points(0,14,pch=21,bg="black",cex=3)
text(0.002,14,"Generalists",cex=1.5,pos=4)
points(0,13.5,pch=22,bg="black",cex=3)
text(0.002,13.5,"Dead Leaf",cex=1.5,pos=4)
points(0,13,pch=23,bg="black",cex=3)
text(0.002,13,"Flower",cex=1.5,pos=4)
points(0,12.5,pch=24,bg="black",cex=3)
text(0.002,12.5,"Stick",cex=1.5,pos=4)


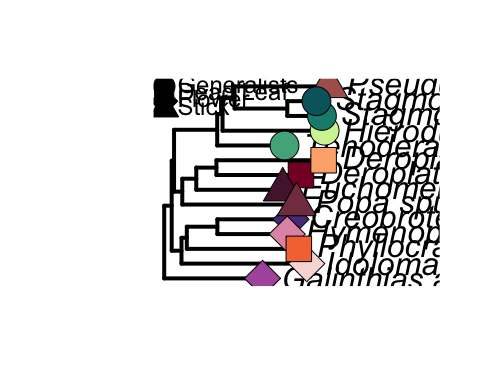


#dev.off()

# Repeatability

ID.ig<-ICCest(data$ID,data$lg.IG,CI.type="THD")
ID.rt<-ICCest(data$ID,data$lg.time,CI.type="THD")
ID.hp<-ICCest(data$ID,log10(data$H.prey.mass.s),CI.type="THD")
#Ingestion rate repeatability
print(ID.ig)

## $ICC
## [1] 0.7761605
##
## $LowerCI
## [1] 0.7010876
##
## $UpperCI
## [1] 0.8419725
##
## $N
## [1] 66
##
## $k
## [1] 4.905413
##
## $varw
## [1] 0.0325266
##
## $vara
## [1] 0.1127855

#ingestion time repeatability
print(ID.rt)

## $ICC
## [1] 0.8520978
##
## $LowerCI
## [1] 0.7974968
##
## $UpperCI
## [1] 0.8977938
##
## $N
## [1] 66
##
## $k
## [1] 4.905413
##
## $varw
## [1] 0.01776062
##
## $vara
## [1] 0.1023229

#prey-mass specific handling time repeatability
print(ID.hp)

## $ICC
## [1] 0.7931576
##
## $LowerCI
## [1] 0.7222514
##
## $UpperCI
## [1] 0.8546758
##
## $N
## [1] 66
##
## $k
## [1] 4.905413
##
## $varw
## [1] 0.02756233
##
## $vara
## [1] 0.1056905

# Allometry models

## Data cleanup and aggregation

#aggregate by individual
inv.phylo<-inverseA(tree.1,nodes="TIPS",scale=FALSE)
prior2<-list(G=list(G1=list(V=1,nu=0.02),G2=list(V=1,nu=0.02)),
 R=list(V=1,nu=0.02))

data.2<-aggregate(data[,tr], by=list(data$Scientific.Name,data$phylo,data$ID),
FUN=mean, na.rm=TRUE)

colnames(data.2)[1] ="species"
colnames(data.2)[2] ="phylo"

dcol<-hcl.colors(6, palette="OrRd",alpha=0.4)
fcol<-hcl.colors(4, palette="PurpOr",alpha=0.4)
scol<-hcl.colors(6, palette="BrwnYl",alpha=0.4)
gcol<-hcl.colors(6, palette="Emrld",alpha=0.4)
len<-nrow(data.2)
for (i in 1:len){
 if(data.2$species[i]=="Creobroter gemmatus"){data.2$col[i]<-fcol[1]}
 if(data.2$species[i]=="Deroplatys dessicata"){data.2$col[i]<-dcol[1]}
 if(data.2$species[i]=="Deroplatys truncata"){data.2$col[i]<-dcol[4]}
 if(data.2$species[i]=="Euchomenlla heteroptera"){data.2$col[i]<-scol[1]}
 if(data.2$species[i]=="Galinthias ameona"){data.2$col[i]<-fcol[2]}
 if(data.2$species[i]=="Hierodula membranacea"){data.2$col[i]<-gcol[6]}
 if(data.2$species[i]=="Hymenopus coronatus"){data.2$col[i]<-fcol[3]}
 if(data.2$species[i]=="Idolomantis diabolica" ){data.2$col[i]<-fcol[4]}
 if(data.2$species[i]=="Phyllocrania paradoxa"){data.2$col[i]<-dcol[3]}
 if(data.2$species[i]=="Popa spurca"){data.2$col[i]<-scol[2]}
 if(data.2$species[i]=="Pseudovates chlorophea"){data.2$col[i]<-scol[3]}
 if(data.2$species[i]=="Sonora Tiger"){data.2$col[i]<-gcol[2]}
 if(data.2$species[i]=="Stagomantis limbata"){data.2$col[i]<-gcol[3]}
 if(data.2$species[i]=="Tenodera sinensis"){data.2$col[i]<-gcol[4]}
 }

len<-nrow(data.2)
for (i in 1:len){
 if(data.2$species[i]=="Creobroter gemmatus"){data.2$sh[i]<-23}
 if(data.2$species[i]=="Deroplatys dessicata"){data.2$sh[i]<-22}
 if(data.2$species[i]=="Deroplatys truncata"){data.2$sh[i]<-22}
 if(data.2$species[i]=="Euchomenlla heteroptera"){data.2$sh[i]<-24}
 if(data.2$species[i]=="Galinthias ameona"){data.2$sh[i]<-23}
 if(data.2$species[i]=="Hierodula membranacea"){data.2$sh[i]<-21}
 if(data.2$species[i]=="Hymenopus coronatus"){data.2$sh[i]<-23}
 if(data.2$species[i]=="Idolomantis diabolica" ){data.2$sh[i]<-23}
 if(data.2$species[i]=="Phyllocrania paradoxa"){data.2$sh[i]<-22}
 if(data.2$species[i]=="Popa spurca"){data.2$sh[i]<-24}
 if(data.2$species[i]=="Pseudovates chlorophea"){data.2$sh[i]<-24}
 if(data.2$species[i]=="Sonora Tiger"){data.2$sh[i]<-21}
 if(data.2$species[i]=="Stagomantis limbata"){data.2$sh[i]<-21}
 if(data.2$species[i]=="Tenodera sinensis"){data.2$sh[i]<-21}
 }

for (i in 1:len){
 if(data.2$species[i]=="Creobroter gemmatus"){data.2$Ecol[i]<-"flower"}
 if(data.2$species[i]=="Deroplatys dessicata"){data.2$Ecol[i]<-"dead leaf"}
 if(data.2$species[i]=="Deroplatys truncata"){data.2$Ecol[i]<-"dead leaf"}
 if(data.2$species[i]=="Euchomenlla heteroptera"){data.2$Ecol[i]<-"stick"}
 if(data.2$species[i]=="Galinthias ameona"){data.2$Ecol[i]<-"flower"}
 if(data.2$species[i]=="Hierodula membranacea"){data.2$Ecol[i]<-"generalist"}
 if(data.2$species[i]=="Hymenopus coronatus"){data.2$Ecol[i]<-"flower"}
 if(data.2$species[i]=="Idolomantis diabolica" ){data.2$Ecol[i]<-"flower"}
 if(data.2$species[i]=="Phyllocrania paradoxa"){data.2$Ecol[i]<-"dead leaf"}
 if(data.2$species[i]=="Popa spurca"){data.2$Ecol[i]<-"stick"}
 if(data.2$species[i]=="Pseudovates chlorophea"){data.2$Ecol[i]<-"stick"}
 if(data.2$species[i]=="Sonora Tiger"){data.2$Ecol[i]<-"generalist"}
 if(data.2$species[i]=="Stagomantis limbata"){data.2$Ecol[i]<-"generalist"}
 if(data.2$species[i]=="Tenodera sinensis"){data.2$Ecol[i]<-"generalist"}
 }

data.2$spec_mean_lg.mass<-sapply(split(data.2$lg.mass,data.2$phylo),mean)[data.2$phylo]
data.2$within_spec_lg.mass<-data.2$lg.mass-data.2$spec_mean_lg.mass

data.2$spec_mean_lg.prey.pred<-sapply(split(data.2$lg.prey.pred,data.2$phylo),mean)[data.2$phylo]
data.2$within_spec_lg.prey.pred<-data.2$lg.prey.pred-data.2$spec_mean_lg.prey.pred


data.2$spec_mean_lg.pred.prey<-sapply(split(data.2$lg.pred.prey,data.2$phylo),mean)[data.2$phylo]
data.2$within_spec_lg.pred.prey<-data.2$lg.pred.prey-data.2$spec_mean_lg.pred.prey

## Group 1: Ingestion rate vs. predator mass

### Phylogenetic general linear mixed model (PGLMM)

With and without the effect of camouflage Note: because these are MCMCglmm models, repeat runs will produce qualitatively, but not necessarily quantiatively similar results.

#PGLMM with camouflage
model_IGE<-MCMCglmm(lg.IG~spec_mean_lg.mass+within_spec_lg.mass+Ecol,
 random=~phylo+species,family="gaussian",
 ginverse=list(phylo=inv.phylo$Ainv),prior=prior2,data=data.2,
 scale=TRUE,verbose=FALSE,
 nitt=1000000,burnin=1000,thin=500)
 summary(model_IGE)

##
## Iterations = 1001:999501
## Thinning interval = 500
## Sample size = 1998
##
## DIC: -33.81795
##
## G-structure: ~phylo
##
## post.mean l-95% CI u-95% CI eff.samp
## phylo 0.08768 0.001492 0.3095 1998
##
## ~species
##
## post.mean l-95% CI u-95% CI eff.samp
## species 0.01271 0.001814 0.03184 1998
##
## R-structure: ~units
##
## post.mean l-95% CI u-95% CI eff.samp
## units 0.02958 0.01908 0.04071 1998
##
## Location effects: lg.IG ~ spec_mean_lg.mass + within_spec_lg.mass + Ecol
##
## post.mean l-95% CI u-95% CI eff.samp pMCMC
## (Intercept) -1.202878 -1.381809 -0.995588 1998 <5e-04 ***
## spec_mean_lg.mass 0.917082 0.635711 1.167465 1998 <5e-04 ***
## within_spec_lg.mass 0.237074 -0.243395 0.677787 1998 0.3043
## Ecolflower 0.263219 0.008359 0.533446 1998 0.0501 .
## Ecolgeneralist 0.299301 0.036639 0.544339 1998 0.0300 *
## Ecolstick 0.187279 -0.057774 0.456639 2142 0.1481
## ---
## Signif. codes: 0 '***' 0.001 '**' 0.01 '*' 0.05 '.' 0.1 ' ' 1

m<-emmeans(model_IGE,specs=~Ecol,data=data.2)
summary(pairs(m),freq=TRUE)

## contrast estimate SE df z.ratio p.value
## dead leaf - flower -0.2632 0.136 Inf -1.935 0.2134
## dead leaf - generalist -0.2993 0.130 Inf -2.298 0.0984
## dead leaf - stick -0.1873 0.131 Inf -1.435 0.4774
## flower - generalist -0.0361 0.130 Inf -0.277 0.9926
## flower - stick 0.0759 0.123 Inf 0.616 0.9269
## generalist - stick 0.1120 0.124 Inf 0.905 0.8019
##
## P value adjustment: tukey method for comparing a family of 4 estimates

#PGLMM without camouflage
model_IG<-MCMCglmm(lg.IG~spec_mean_lg.mass+within_spec_lg.mass,
 random=~phylo+species,family="gaussian",
 ginverse=list(phylo=inv.phylo$Ainv),prior=prior2,data=data.2,
 scale=TRUE, verbose=FALSE,
 nitt=1000000,burnin=1000,thin=500)
 summary(model_IG)

##
## Iterations = 1001:999501
## Thinning interval = 500
## Sample size = 1998
##
## DIC: -32.78329
##
## G-structure: ~phylo
##
## post.mean l-95% CI u-95% CI eff.samp
## phylo 0.15 0.002287 0.4799 1998
##
## ~species
##
## post.mean l-95% CI u-95% CI eff.samp
## species 0.01592 0.00203 0.03889 1981
##
## R-structure: ~units
##
## post.mean l-95% CI u-95% CI eff.samp
## units 0.03017 0.01914 0.04219 1998
##
## Location effects: lg.IG ~ spec_mean_lg.mass + within_spec_lg.mass
##
## post.mean l-95% CI u-95% CI eff.samp pMCMC
## (Intercept) -1.0038 -1.1163 -0.8941 1998 <5e-04 ***
## spec_mean_lg.mass 0.8706 0.5912 1.1441 1998 <5e-04 ***
## within_spec_lg.mass 0.2125 -0.2493 0.6860 1998 0.381
## ---
## Signif. codes: 0 '***' 0.001 '**' 0.01 '*' 0.05 '.' 0.1 ' ' 1

lambda <- model_IG$VCV[,'phylo']/
 (model_IG$VCV[,'phylo']+model_IG$VCV[,'species']+
 model_IG$VCV[,'units'])
mean(lambda)

## [1] 0.6308911

AICc(model_IGE,model_IG)#model without camouflage better

## df AICc
## model_IGE 9 -25.88099
## model_IG 6 -32.05642

### Phylogenetic Generalised Least Squares (PGLS)

With and without the effect of camouflage

df.phylo<-comparative.data(tree.1, data.1, Tip.label, vcv=TRUE, vcv.dim=3)

#with camouflage
mod.IG.E<-pgls(lg.IG~lg.mass+Ecol , df.phylo, lambda='ML')
summary(mod.IG.E)

##
## Call:
## pgls(formula = lg.IG ~ lg.mass + Ecol, data = df.phylo, lambda = "ML")
##
## Residuals:
## Min 1Q Median 3Q Max
## -0.64944 -0.08895 0.06238 0.08357 0.59048
##
## Branch length transformations:
##
## kappa [Fix] : 1.000
## lambda [ ML] : 1.000
## lower bound : 0.000, p = 0.46056
## upper bound : 1.000, p = 1
## 95.0% CI : (NA, NA)
## delta [Fix] : 1.000
##
## Coefficients:
## Estimate Std. Error t value Pr(>|t|)
## (Intercept) -1.186150 0.071773 -16.5265 4.849e-08 ***
## lg.mass 0.923720 0.076872 12.0164 7.611e-07 ***
## Ecolflower 0.175753 0.080733 2.1770 0.057459 .
## Ecolgeneralist 0.316709 0.100317 3.1571 0.011604 *
## Ecolstick 0.331268 0.100765 3.2875 0.009416 **
## ---
## Signif. codes: 0 '***' 0.001 '**' 0.01 '*' 0.05 '.' 0.1 ' ' 1
##
## Residual standard error: 0.404 on 9 degrees of freedom
## Multiple R-squared: 0.9483, Adjusted R-squared: 0.9253
## F-statistic: 41.26 on 4 and 9 DF, p-value: 8.561e-06

anova(mod.IG.E)

## Analysis of Variance Table
## Sequential SS for pgls: lambda = 1.00, delta = 1.00, kappa = 1.00
##
## Response: lg.IG
## Df Sum Sq Mean Sq F value Pr(>F)
## lg.mass 1 24.8462 24.8462 152.2647 6.069e-07 ***
## Ecol 3 2.0879 0.6960 4.2651 0.03929 *
## Residuals 9 1.4686 0.1632
## ---
## Signif. codes: 0 '***' 0.001 '**' 0.01 '*' 0.05 '.' 0.1 ' ' 1

#without camouflage
mod.IG<-pgls(lg.IG~lg.mass , df.phylo, lambda='ML')
summary(mod.IG)

##
## Call:
## pgls(formula = lg.IG ~ lg.mass, data = df.phylo, lambda = "ML")
##
## Residuals:
## Min 1Q Median 3Q Max
## -1.2021 -0.1224 0.1636 0.3636 0.6958
##
## Branch length transformations:
##
## kappa [Fix] : 1.000
## lambda [ ML] : 1.000
## lower bound : 0.000, p = 0.050409
## upper bound : 1.000, p = 1
## 95.0% CI : (NA, NA)
## delta [Fix] : 1.000
##
## Coefficients:
## Estimate Std. Error t value Pr(>|t|)
## (Intercept) -1.006055 0.058784 -17.1145 8.528e-10 ***
## lg.mass 0.923274 0.098726 9.3519 7.350e-07 ***
## ---
## Signif. codes: 0 '***' 0.001 '**' 0.01 '*' 0.05 '.' 0.1 ' ' 1
##
## Residual standard error: 0.5444 on 12 degrees of freedom
## Multiple R-squared: 0.8748, Adjusted R-squared: 0.8643
## F-statistic: 83.83 on 1 and 12 DF, p-value: 9.204e-07

AICc(mod.IG.E,mod.IG)#without camouflage better

## df AICc
## mod.IG.E 5 -13.20184
## mod.IG 2 -13.22837

## Group 2: Ingestion time against predator mass

### PGLMM

With and without the effect of camouflage Note: because these are MCMCglmm models, repeat runs will produce qualitatively, but not necessarily quantiatively similar results.

model_time_mass_E<-MCMCglmm(lg.time~spec_mean_lg.mass+within_spec_lg.mass+Ecol,
 random=~phylo+species,family="gaussian",
 ginverse=list(phylo=inv.phylo$Ainv),prior=prior2,data=data.2,
 scale=TRUE, verbose=FALSE,
 nitt=1000000,burnin=1000,thin=500)
#With camouflage
summary(model_time_mass_E)

##
## Iterations = 1001:999501
## Thinning interval = 500
## Sample size = 1998
##
## DIC: -110.6671
##
## G-structure: ~phylo
##
## post.mean l-95% CI u-95% CI eff.samp
## phylo 0.1247 0.003022 0.4229 1998
##
## ~species
##
## post.mean l-95% CI u-95% CI eff.samp
## species 0.01844 0.001271 0.04411 1998
##
## R-structure: ~units
##
## post.mean l-95% CI u-95% CI eff.samp
## units 0.00928 0.005868 0.01303 1842
##
## Location effects: lg.time ~ spec_mean_lg.mass + within_spec_lg.mass + Ecol
##
## post.mean l-95% CI u-95% CI eff.samp pMCMC
## (Intercept) 2.809807 2.596970 3.017746 1998 <5e-04 ***
## spec_mean_lg.mass -0.874060 -1.145917 -0.605907 1998 <5e-04 ***
## within_spec_lg.mass -0.259031 -0.525217 0.004208 1998 0.0521 .
## Ecolflower -0.216586 -0.481437 0.064110 1998 0.1121
## Ecolgeneralist -0.277532 -0.542928 0.026642 1998 0.0581 .
## Ecolstick -0.234263 -0.506523 0.052479 2583 0.0891 .
## ---
## Signif. codes: 0 '***' 0.001 '**' 0.01 '*' 0.05 '.' 0.1 ' ' 1

m<-emmeans(model_time_mass_E,specs=~Ecol,data=data.2)
summary(m,freq=TRUE)

## Ecol emmean SE df asymp.LCL asymp.UCL
## dead leaf 2.83 0.1059 Inf 2.62 3.04
## flower 2.61 0.0922 Inf 2.43 2.79
## generalist 2.55 0.1040 Inf 2.35 2.76
## stick 2.60 0.1015 Inf 2.40 2.80
##
## Confidence level used: 0.95

summary(pairs(m),freq=TRUE)#no difference among ecomorphs

## contrast estimate SE df z.ratio p.value
## dead leaf - flower 0.2166 0.140 Inf 1.543 0.4115
## dead leaf - generalist 0.2775 0.143 Inf 1.945 0.2092
## dead leaf - stick 0.2343 0.141 Inf 1.658 0.3461
## flower - generalist 0.0609 0.139 Inf 0.438 0.9719
## flower - stick 0.0177 0.136 Inf 0.130 0.9992
## generalist - stick -0.0433 0.137 Inf -0.316 0.9891
##
## P value adjustment: tukey method for comparing a family of 4 estimates

model_time_mass<-MCMCglmm(lg.time~spec_mean_lg.mass+within_spec_lg.mass,
 random=~phylo+species,family="gaussian",
 ginverse=list(phylo=inv.phylo$Ainv),prior=prior2,data=data.2,
 scale=TRUE, verbose=FALSE,
 nitt=1000000,burnin=1000,thin=500)
#Without camouflage
summary(model_time_mass)

##
## Iterations = 1001:999501
## Thinning interval = 500
## Sample size = 1998
##
## DIC: -110.6102
##
## G-structure: ~phylo
##
## post.mean l-95% CI u-95% CI eff.samp
## phylo 0.172 0.002236 0.518 1998
##
## ~species
##
## post.mean l-95% CI u-95% CI eff.samp
## species 0.01897 0.001798 0.04591 1790
##
## R-structure: ~units
##
## post.mean l-95% CI u-95% CI eff.samp
## units 0.009297 0.006177 0.01354 1998
##
## Location effects: lg.time ~ spec_mean_lg.mass + within_spec_lg.mass
##
## post.mean l-95% CI u-95% CI eff.samp pMCMC
## (Intercept) 2.62548 2.50383 2.73852 1998 <5e-04 ***
## spec_mean_lg.mass -0.83591 -1.07711 -0.56863 2167 <5e-04 ***
## within_spec_lg.mass -0.26213 -0.52025 -0.00434 1998 0.046 *
## ---
## Signif. codes: 0 '***' 0.001 '**' 0.01 '*' 0.05 '.' 0.1 ' ' 1

#AICc
 AICc(model_time_mass_E,model_time_mass)#model without camouflage better

## df AICc
## model_time_mass_E 9 -104.0662
## model_time_mass 6 -112.0467

#Lambda of model without camouflage
 lambda <- model_time_mass$VCV[,'phylo']/
 (model_time_mass$VCV[,'phylo']+model_time_mass$VCV[,'species']+
 model_time_mass$VCV[,'units'])
mean(lambda)

## [1] 0.7337716

### PGLS

With and without the effect of camouflage

#with camouflage
mod.time.mass.E<-pgls(lg.time~lg.mass+Ecol , df.phylo, lambda='ML')
summary(mod.time.mass.E)

##
## Call:
## pgls(formula = lg.time ~ lg.mass + Ecol, data = df.phylo, lambda = "ML")
##
## Residuals:
## Min 1Q Median 3Q Max
## -0.46033 -0.18930 -0.03406 0.11781 0.66866
##
## Branch length transformations:
##
## kappa [Fix] : 1.000
## lambda [ ML] : 0.000
## lower bound : 0.000, p = 1
## upper bound : 1.000, p = 0.2048
## 95.0% CI : (NA, NA)
## delta [Fix] : 1.000
##
## Coefficients:
## Estimate Std. Error t value Pr(>|t|)
## (Intercept) 2.807724 0.060534 46.3823 5.038e-12 ***
## lg.mass -0.840074 0.074032 -11.3475 1.238e-06 ***
## Ecolflower -0.167393 0.077525 -2.1592 0.059142 .
## Ecolgeneralist -0.286183 0.079248 -3.6112 0.005649 **
## Ecolstick -0.320054 0.095707 -3.3441 0.008604 **
## ---
## Signif. codes: 0 '***' 0.001 '**' 0.01 '*' 0.05 '.' 0.1 ' ' 1
##
## Residual standard error: 0.3721 on 9 degrees of freedom
## Multiple R-squared: 0.9494, Adjusted R-squared: 0.9269
## F-statistic: 42.18 on 4 and 9 DF, p-value: 7.803e-06

anova(mod.time.mass.E)

## Analysis of Variance Table
## Sequential SS for pgls: lambda = 0.00, delta = 1.00, kappa = 1.00
##
## Response: lg.time
## Df Sum Sq Mean Sq F value Pr(>F)
## lg.mass 1 21.0244 21.0244 151.8360 6.142e-07 ***
## Ecol 3 2.3376 0.7792 5.6274 0.01885 *
## Residuals 9 1.2462 0.1385
## ---
## Signif. codes: 0 '***' 0.001 '**' 0.01 '*' 0.05 '.' 0.1 ' ' 1

#without camouflage
mod.time.mass<-pgls(lg.time~lg.mass , df.phylo, lambda='ML')
summary(mod.time.mass)

##
## Call:
## pgls(formula = lg.time ~ lg.mass, data = df.phylo, lambda = "ML")
##
## Residuals:
## Min 1Q Median 3Q Max
## -0.8012 -0.3439 -0.1561 0.1078 1.1656
##
## Branch length transformations:
##
## kappa [Fix] : 1.000
## lambda [ ML] : 1.000
## lower bound : 0.000, p = 0.10838
## upper bound : 1.000, p = 1
## 95.0% CI : (NA, NA)
## delta [Fix] : 1.000
##
## Coefficients:
## Estimate Std. Error t value Pr(>|t|)
## (Intercept) 2.643755 0.058504 45.1895 8.882e-15 ***
## lg.mass -0.868706 0.098255 -8.8413 1.332e-06 ***
## ---
## Signif. codes: 0 '***' 0.001 '**' 0.01 '*' 0.05 '.' 0.1 ' ' 1
##
## Residual standard error: 0.5418 on 12 degrees of freedom
## Multiple R-squared: 0.8621, Adjusted R-squared: 0.8506
## F-statistic: 75.02 on 1 and 12 DF, p-value: 1.653e-06

AICc(mod.time.mass.E, mod.time.mass)#without camouflage better

## df AICc
## mod.time.mass.E 5 -13.16410
## mod.time.mass 2 -13.36215

## Group 3: Prey-mass specific handling time (handling time (s)/prey mass (mg) against predator mass

### PGLMM

With and without the effect of camouflage Note: because these are MCMCglmm models, repeat runs will produce qualitatively, but not necessarily quantiatively similar results.

model_time_pdpy_E<-MCMCglmm(log10(H.prey.mass.s)~spec_mean_lg.mass+within_spec_lg.mass+Ecol,
 random=~phylo+species,family="gaussian",
 ginverse=list(phylo=inv.phylo$Ainv),prior=prior2,data=data.2,
 scale=TRUE, verbose=FALSE,
 nitt=1000000,burnin=1000,thin=500)
#with camouflage
 summary(model_time_pdpy_E)

##
## Iterations = 1001:999501
## Thinning interval = 500
## Sample size = 1998
##
## DIC: -31.40661
##
## G-structure: ~phylo
##
## post.mean l-95% CI u-95% CI eff.samp
## phylo 0.08628 0.001956 0.3272 1998
##
## ~species
##
## post.mean l-95% CI u-95% CI eff.samp
## species 0.01294 0.001572 0.03223 1998
##
## R-structure: ~units
##
## post.mean l-95% CI u-95% CI eff.samp
## units 0.03104 0.02037 0.04376 1998
##
## Location effects: log10(H.prey.mass.s) ~ spec_mean_lg.mass + within_spec_lg.mass + Ecol
##
## post.mean l-95% CI u-95% CI eff.samp pMCMC
## (Intercept) 1.236566 1.036071 1.425625 1998 <5e-04 ***
## spec_mean_lg.mass -0.911858 -1.145398 -0.642894 1998 <5e-04 ***
## within_spec_lg.mass -0.390537 -0.899648 0.075419 1998 0.121
## Ecolflower -0.273243 -0.528794 -0.005525 1998 0.044 *
## Ecolgeneralist -0.290996 -0.542290 -0.029332 1966 0.030 *
## Ecolstick -0.194722 -0.453092 0.059873 1998 0.130
## ---
## Signif. codes: 0 '***' 0.001 '**' 0.01 '*' 0.05 '.' 0.1 ' ' 1

m<-emmeans(model_time_pdpy_E,specs=~Ecol,data=data.2)
summary(m,freq=TRUE)

## Ecol emmean SE df asymp.LCL asymp.UCL
## dead leaf 1.258 0.0987 Inf 1.065 1.45
## flower 0.985 0.0916 Inf 0.806 1.16
## generalist 0.967 0.0956 Inf 0.780 1.15
## stick 1.063 0.0956 Inf 0.876 1.25
##
## Confidence level used: 0.95

summary(pairs(m),freq=TRUE)#no difference among ecomorphs

## contrast estimate SE df z.ratio p.value
## dead leaf - flower 0.2732 0.138 Inf 1.986 0.1934
## dead leaf - generalist 0.2910 0.129 Inf 2.253 0.1093
## dead leaf - stick 0.1947 0.131 Inf 1.488 0.4447
## flower - generalist 0.0178 0.137 Inf 0.130 0.9992
## flower - stick -0.0785 0.128 Inf -0.611 0.9286
## generalist - stick -0.0963 0.128 Inf -0.754 0.8748
##
## P value adjustment: tukey method for comparing a family of 4 estimates

model_time_pdpy<-MCMCglmm(log10(H.prey.mass.s)~spec_mean_lg.mass+within_spec_lg.mass,
 random=~phylo+species,family="gaussian",
 ginverse=list(phylo=inv.phylo$Ainv),prior=prior2,data=data.2,
 scale=TRUE, verbose=FALSE,
 nitt=1000000,burnin=1000,thin=500)
#without camouflage
 summary(model_time_pdpy)

##
## Iterations = 1001:999501
## Thinning interval = 500
## Sample size = 1998
##
## DIC: -30.43725
##
## G-structure: ~phylo
##
## post.mean l-95% CI u-95% CI eff.samp
## phylo 0.1313 0.002374 0.4274 1998
##
## ~species
##
## post.mean l-95% CI u-95% CI eff.samp
## species 0.0162 0.001737 0.04136 1998
##
## R-structure: ~units
##
## post.mean l-95% CI u-95% CI eff.samp
## units 0.03128 0.02005 0.04384 1998
##
## Location effects: log10(H.prey.mass.s) ~ spec_mean_lg.mass + within_spec_lg.mass
##
## post.mean l-95% CI u-95% CI eff.samp pMCMC
## (Intercept) 1.0358 0.9239 1.1530 1998 <5e-04 ***
## spec_mean_lg.mass -0.8641 -1.1241 -0.6045 1998 <5e-04 ***
## within_spec_lg.mass -0.3751 -0.8756 0.1194 1998 0.133
## ---
## Signif. codes: 0 '***' 0.001 '**' 0.01 '*' 0.05 '.' 0.1 ' ' 1

#AICc of models
 AICc(model_time_pdpy_E,model_time_pdpy)#model without camouflage better

## df AICc
## model_time_pdpy_E 9 -22.79352
## model_time_pdpy 6 -29.64783

#lambda of model without camouflage
 lambda <- model_time_pdpy$VCV[,'phylo']/
 (model_time_pdpy$VCV[,'phylo']+model_time_pdpy$VCV[,'species']+
 model_time_pdpy$VCV[,'units'])
mean(lambda)

## [1] 0.5959568

### PGLS

With and without the effect of camouflage

#with camouflage
#note, lambda had to be set to 1, which it comes out as anyway, because it gives an optimization error in R studio if it is set to ML
#this problem is does not happen in base R
mod.time.E<-pgls(log10(H.prey.mass.s)~lg.mass+Ecol, df.phylo, lambda=1)
summary(mod.time.E)

##
## Call:
## pgls(formula = log10(H.prey.mass.s) ~ lg.mass + Ecol, data = df.phylo,
## lambda = 1)
##
## Residuals:
## Min 1Q Median 3Q Max
## -0.70458 -0.21869 -0.00004 0.14488 0.67892
##
## Branch length transformations:
##
## kappa [Fix] : 1.000
## lambda [Fix] : 1.000
## delta [Fix] : 1.000
##
## Coefficients:
## Estimate Std. Error t value Pr(>|t|)
## (Intercept) 1.240430 0.078841 15.7332 7.448e-08 ***
## lg.mass -0.957943 0.084442 -11.3443 1.241e-06 ***
## Ecolflower -0.211889 0.088685 -2.3892 0.04061 *
## Ecolgeneralist -0.273601 0.110197 -2.4828 0.03483 *
## Ecolstick -0.306474 0.110689 -2.7688 0.02180 *
## ---
## Signif. codes: 0 '***' 0.001 '**' 0.01 '*' 0.05 '.' 0.1 ' ' 1
##
## Residual standard error: 0.4437 on 9 degrees of freedom
## Multiple R-squared: 0.9401, Adjusted R-squared: 0.9135
## F-statistic: 35.32 on 4 and 9 DF, p-value: 1.647e-05

anova(mod.time.E)

## Analysis of Variance Table
## Sequential SS for pgls: lambda = 1.00, delta = 1.00, kappa = 1.00
##
## Response: log10(H.prey.mass.s)
## Df Sum Sq Mean Sq F value Pr(>F)
## lg.mass 1 25.9707 25.9707 131.8965 1.118e-06 ***
## Ecol 3 1.8461 0.6154 3.1252 0.0805 .
## Residuals 9 1.7721 0.1969
## ---
## Signif. codes: 0 '***' 0.001 '**' 0.01 '*' 0.05 '.' 0.1 ' ' 1

#without camouflage
mod.time<-pgls(log10(H.prey.mass.s)~lg.mass, df.phylo, lambda='ML')
summary(mod.time)

##
## Call:
## pgls(formula = log10(H.prey.mass.s) ~ lg.mass, data = df.phylo,
## lambda = "ML")
##
## Residuals:
## Min 1Q Median 3Q Max
## -0.77956 -0.38847 -0.12747 0.09259 1.25501
##
## Branch length transformations:
##
## kappa [Fix] : 1.000
## lambda [ ML] : 1.000
## lower bound : 0.000, p = 0.068844
## upper bound : 1.000, p = 1
## 95.0% CI : (NA, NA)
## delta [Fix] : 1.000
##
## Coefficients:
## Estimate Std. Error t value Pr(>|t|)
## (Intercept) 1.055822 0.059292 17.8073 5.387e-10 ***
## lg.mass -0.941582 0.099579 -9.4557 6.534e-07 ***
## ---
## Signif. codes: 0 '***' 0.001 '**' 0.01 '*' 0.05 '.' 0.1 ' ' 1
##
## Residual standard error: 0.5491 on 12 degrees of freedom
## Multiple R-squared: 0.8777, Adjusted R-squared: 0.8675
## F-statistic: 86.13 on 1 and 12 DF, p-value: 7.972e-07

#AICc
AICc(mod.time.E,mod.time)#without ecology slightly better

## df AICc
## mod.time.E 5 -10.57171
## mod.time 2 -12.98756

# Figure 2

## Figure 2A ingestion rate vs. predator mass

Larger points are species means, smaller points individual means PGLS regression line in gray dash PGLS confidence intervals in gray dot PGLMM regression line in solid black

#to save figure as png file use the code below
#png(file="Ingestion rate allometry log10.png", height=12, width=12, units="in",res=300)
par(mar=c(5.1,6,4.1,2.1))
plot(data.2$lg.mass,data.2$lg.IG,pch=data.2$sh,bg=data.2$col,col=data.2$col,cex=2,xlab=expression(log[10]~Predator~Mass~(g)),ylab=expression(log[10]~Ingestion~Rate~(mg~s^-1)),cex.lab=2,cex.axis=2,axes=FALSE,xlim=c(-1,1),ylim=c(-2,0))
Axis(side=1, labels=TRUE,cex.axis=2)
Axis(side=2, labels=TRUE,cex.axis=2)
points(data.1$lg.mass,data.1$lg.IG,pch=data.1$sh,bg=data.1$col,cex=4)
abline(a=-1.006055,b=0.92327,col="darkgray",lty=2,lwd=4)#pgls
abline(a=mean(model_IG$Sol[,1]),b=mean(model_IG$Sol[,2]),col="black",lwd=4)
pGLS_ci<-gls.ci(data.1$lg.IG,data.1$lg.mass,vcv(tree.1))#note run function below first
 lines(pGLS_ci$CI.plot$X,pGLS_ci$CI.plot$Lower5,lty=3,col="darkgray",lwd=3)
 lines(pGLS_ci$CI.plot$X,pGLS_ci$CI.plot$Upper5,lty=3,col="darkgray",lwd=3)

 points(-1,0,cex=2,pch=21,bg=data.1$col[14])
 points(-1,-0.05,cex=2,pch=21,bg=data.1$col[13])
 points(-1,-0.1,cex=2,pch=21,bg=data.1$col[12])
 points(-1,-0.15,cex=2,pch=21,bg=data.1$col[6])
 points(-1,-0.2,cex=2,pch=23,bg=data.1$col[1])
 points(-1,-0.25,cex=2,pch=23,bg=data.1$col[5])
 points(-1,-0.3,cex=2,pch=23,bg=data.1$col[7])
 points(-1,-0.35,cex=2,pch=23,bg=data.1$col[8])
 points(-1,-0.4,cex=2,pch=22,bg=data.1$col[2])
 points(-1,-0.45,cex=2,pch=22,bg=data.1$col[3])
 points(-1,-0.5,cex=2,pch=22,bg=data.1$col[9])
 points(-1,-0.55,cex=2,pch=24,bg=data.1$col[4])
 points(-1,-0.6,cex=2,pch=24,bg=data.1$col[10])
 points(-1,-0.65,cex=2,pch=24,bg=data.1$col[11])
 text(x=-1,y=0, label="Tenodera sinensis",pos=4,offset=0.75,cex=1)
 text(x=-1,y=-0.05, label="Stagmomantis limbata",offset=0.75,pos=4,cex=1)
 text(x=-1,y=-0.1, label="Stagmomantis clauseni",pos=4,cex=1,offset=0.75)
 text(x=-1,y=-0.15, label="Hierodula membranacea",pos=4,cex=1,offset=0.75)
 text(x=-1,y=-0.2, label="Creobroter gemmatus",pos=4,cex=1,offset=0.75)
 text(x=-1,y=-0.25, label="Galinthias ameona",pos=4,cex=1,offset=0.75)
 text(x=-1,y=-0.3, label="Hymenopus coronatus",pos=4,cex=1,offset=0.75)
 text(x=-1,y=-0.35, label="Idolomantis diabolica",pos=4,cex=1,offset=0.75)
 text(x=-1,y=-0.4, label="Deroplatys desiccata",pos=4,cex=1,offset=0.75)
 text(x=-1,y=-0.45, label="Deroplatys truncata",pos=4,cex=1,offset=0.75)
 text(x=-1,y=-0.5, label="Phyllocrania paradoxa",pos=4,cex=1,offset=0.75)
 text(x=-1,y=-0.55, label="Euchomenella heteroptera",pos=4,cex=1,offset=0.75)
 text(x=-1,y=-0.6, label="Popa spurca",pos=4,cex=1,offset=0.75)
 text(x=-1,y=-0.65, label="Pseudovates chlorophea",pos=4,cex=1,offset=0.75)


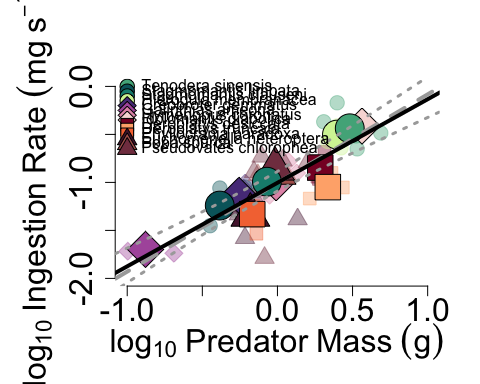


# dev.off()

## Figure 2B ingestion time vs. predator mass

Larger points are species means, smaller points individual means PGLS regression line in gray dash PGLS confidence intervals in gray dot PGLMM regression line in solid black

#to save as png use code below
#png(file="Ingestion time allometry predator mass log10.png", height=12, width=12, units="in",res=300)
par(mar=c(5.1,6,4.1,2.1))
plot(data.2$lg.mass,data.2$lg.time,pch=data.2$sh,bg=data.2$col,col=data.2$col,cex=2,xlab=expression(log[10]~Predator~Mass~(g)),ylab=expression(log[10]~Ingestion~Time~(s)),cex.lab=2,cex.axis=2,axes=FALSE,xlim=c(-1,1),ylim=c(2,3.5))
Axis(side=1, labels=TRUE,cex.axis=2)
Axis(side=2, labels=TRUE,cex.axis=2)
points(data.1$lg.mass,data.1$lg.time,pch=data.1$sh,bg=data.1$col,cex=4)
abline(a=2.643755,b=-0.868706,col="darkgray",lty=2,lwd=4)#pgls
abline(a=mean(model_time_mass$Sol[,1]),b=mean(model_time_mass$Sol[,2]),col="black",lwd=4)
pGLS_ci<-gls.ci(data.1$lg.time,data.1$lg.mass,vcv(tree.1))#note run function below first
 lines(pGLS_ci$CI.plot$X,pGLS_ci$CI.plot$Lower5,lty=3,col="darkgray",lwd=3)
 lines(pGLS_ci$CI.plot$X,pGLS_ci$CI.plot$Upper5,lty=3,col="darkgray",lwd=3)


 points(-1,2.75,cex=2,pch=21,bg=data.1$col[14])
 points(-1,2.7,cex=2,pch=21,bg=data.1$col[13])
 points(-1,2.65,cex=2,pch=21,bg=data.1$col[12])
 points(-1,2.6,cex=2,pch=21,bg=data.1$col[6])
 points(-1,2.55,cex=2,pch=23,bg=data.1$col[1])
 points(-1,2.5,cex=2,pch=23,bg=data.1$col[5])
 points(-1,2.45,cex=2,pch=23,bg=data.1$col[7])
 points(-1,2.4,cex=2,pch=23,bg=data.1$col[8])
 points(-1,2.35,cex=2,pch=22,bg=data.1$col[2])
 points(-1,2.3,cex=2,pch=22,bg=data.1$col[3])
 points(-1,2.25,cex=2,pch=22,bg=data.1$col[9])
 points(-1,2.2,cex=2,pch=24,bg=data.1$col[4])
 points(-1,2.15,cex=2,pch=24,bg=data.1$col[10])
 points(-1,2.1,cex=2,pch=24,bg=data.1$col[11])
 text(x=-1,y=2.75, label="Tenodera sinensis",pos=4,offset=0.75,cex=1)
 text(x=-1,y=2.7, label="Stagmomantis limbata",offset=0.75,pos=4,cex=1)
 text(x=-1,y=2.65, label="Stagmomantis clauseni",pos=4,cex=1,offset=0.75)
 text(x=-1,y=2.6, label="Hierodula membranacea",pos=4,cex=1,offset=0.75)
 text(x=-1,y=2.55, label="Creobroter gemmatus",pos=4,cex=1,offset=0.75)
 text(x=-1,y=2.5, label="Galinthias ameona",pos=4,cex=1,offset=0.75)
 text(x=-1,y=2.45, label="Hymenopus coronatus",pos=4,cex=1,offset=0.75)
 text(x=-1,y=2.4, label="Idolomantis diabolica",pos=4,cex=1,offset=0.75)
 text(x=-1,y=2.35, label="Deroplatys desiccata",pos=4,cex=1,offset=0.75)
 text(x=-1,y=2.3, label="Deroplatys truncata",pos=4,cex=1,offset=0.75)
 text(x=-1,y=2.25, label="Phyllocrania paradoxa",pos=4,cex=1,offset=0.75)
 text(x=-1,y=2.2, label="Euchomenella heteroptera",pos=4,cex=1,offset=0.75)
 text(x=-1,y=2.15, label="Popa spurca",pos=4,cex=1,offset=0.75)
 text(x=-1,y=2.1, label="Pseudovates chlorophea",pos=4,cex=1,offset=0.75)


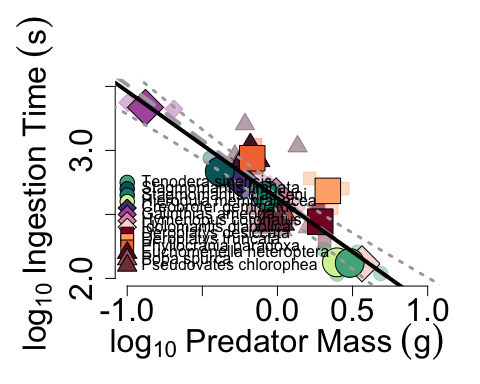


#dev.off()

## Figure 2C prey-mass specific handling time (handling time (s)/prey mass (mg)vs. predator mass

Larger points are species means, smaller points individual means PGLS regression line in gray dash PGLS confidence intervals in gray dot PGLMM regression line in solid black

#to save figure as png use code below
#png(file="Ingestion time allometry log10.png", height=10, width=10, units="in",res=300)
par(mar=c(5.1,6,4.1,2.1))
plot(data.2$lg.mass,log10(data.2$H.prey.mass.s),pch=data.2$sh,bg=data.2$col,col=data.2$col,cex=2,xlab=expression(log[10]~Predator~Mass~(g)),ylab=expression(log[10]~Ingestion~Time/Prey~Mass),cex.lab=2,cex.axis=2,axes=FALSE,xlim=c(-1,1),ylim=c(0.2,1.8))
Axis(side=1, labels=TRUE,cex.axis=2)
Axis(side=2, labels=TRUE,cex.axis=2)
points(data.1$lg.mass,log10(data.1$H.prey.mass.s),pch=data.1$sh,bg=data.1$col,cex=4)
abline(a=1.055822,b=-0.9416,col="darkgray",lty=2,lwd=4)#pgls
abline(a=mean(model_time_pdpy$Sol[,1]),b=mean(model_time_pdpy$Sol[,2]),col="black",lwd=4)
pGLS_ci<-gls.ci(log10(data.1$H.prey.mass.s),data.1$lg.mass,vcv(tree.1))#note run function below first
 lines(pGLS_ci$CI.plot$X,pGLS_ci$CI.plot$Lower5,lty=3,col="darkgray",lwd=3)
 lines(pGLS_ci$CI.plot$X,pGLS_ci$CI.plot$Upper5,lty=3,col="darkgray",lwd=3)

 points(-1,1.1,cex=2,pch=21,bg=data.1$col[14])
 points(-1,1.05,cex=2,pch=21,bg=data.1$col[13])
 points(-1,1,cex=2,pch=21,bg=data.1$col[12])
 points(-1,0.95,cex=2,pch=21,bg=data.1$col[6])
 points(-1,0.9,cex=2,pch=23,bg=data.1$col[1])
 points(-1,0.85,cex=2,pch=23,bg=data.1$col[5])
 points(-1,0.8,cex=2,pch=23,bg=data.1$col[7])
 points(-1,0.75,cex=2,pch=23,bg=data.1$col[8])
 points(-1,0.7,cex=2,pch=22,bg=data.1$col[2])
 points(-1,0.65,cex=2,pch=22,bg=data.1$col[3])
 points(-1,0.6,cex=2,pch=22,bg=data.1$col[9])
 points(-1,0.55,cex=2,pch=24,bg=data.1$col[4])
 points(-1,0.5,cex=2,pch=24,bg=data.1$col[10])
 points(-1,0.45,cex=2,pch=24,bg=data.1$col[11])
 text(x=-1,y=1.1, label="Tenodera sinensis",pos=4,offset=0.75,cex=1)
 text(x=-1,y=1.05, label="Stagmomantis limbata",offset=0.75,pos=4,cex=1)
 text(x=-1,y=1, label="Stagmomantis sp.",pos=4,cex=1,offset=0.75)
 text(x=-1,y=0.95, label="Hierodula membranacea",pos=4,cex=1,offset=0.75)
 text(x=-1,y=0.9, label="Creobroter gemmatus",pos=4,cex=1,offset=0.75)
 text(x=-1,y=0.85, label="Galinthias ameona",pos=4,cex=1,offset=0.75)
 text(x=-1,y=0.8, label="Hymenopus coronatus",pos=4,cex=1,offset=0.75)
 text(x=-1,y=0.75, label="Idolomantis diabolica",pos=4,cex=1,offset=0.75)
 text(x=-1,y=0.7, label="Deroplatys desiccata",pos=4,cex=1,offset=0.75)
 text(x=-1,y=0.65, label="Deroplatys truncata",pos=4,cex=1,offset=0.75)
 text(x=-1,y=0.6, label="Phyllocrania paradoxa",pos=4,cex=1,offset=0.75)
 text(x=-1,y=0.55, label="Euchomenella heteroptera",pos=4,cex=1,offset=0.75)
 text(x=-1,y=0.5, label="Popa spurca",pos=4,cex=1,offset=0.75)
 text(x=-1,y=0.45, label="Pseudovates chlorophea",pos=4,cex=1,offset=0.75)


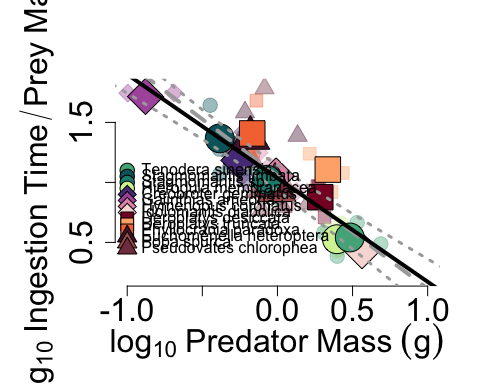


#dev.off()

# Comparisons to FoRAGE dataset

This includes figure 3 and inset 3B Also includes results of regression models in Table 2

## Data clean up

#drop unused levels in truncated data
newdata<-droplevels(newdata)#just insect predators
Mol.data<-droplevels(Mol.data)
Rep.data<-droplevels(Rep.data)
Arach.data<-droplevels(Arach.data)
Mamm.data<-droplevels(Mamm.data)
#remove missing values
newdata<-na.omit(newdata)
Fish.data<-na.omit(Fish.data)
Arach.data<-na.omit(Arach.data)

m<-which(newdata[,1]=="Hierodula crassa")
fcol<-hcl.colors(4, palette="PurpOr")

## Regression models

#Mantis phylogenetic no camouflage
mod.ht<-pgls(log10(H.prey.mass)~log10(Mass.mg) , df.phylo, lambda='ML')
summary(mod.ht)

##
## Call:
## pgls(formula = log10(H.prey.mass) ~ log10(Mass.mg), data = df.phylo,
## lambda = "ML")
##
## Residuals:
## Min 1Q Median 3Q Max
## -0.78564 -0.39663 -0.09593 0.05432 1.25996
##
## Branch length transformations:
##
## kappa [Fix] : 1.000
## lambda [ ML] : 1.000
## lower bound : 0.000, p = 0.067288
## upper bound : 1.000, p = 1
## 95.0% CI : (NA, NA)
## delta [Fix] : 1.000
##
## Coefficients:
## Estimate Std. Error t value Pr(>|t|)
## (Intercept) -1.03011 0.30215 -3.4093 0.00518 **
## log10(Mass.mg) -0.94665 0.10015 -9.4522 6.56e-07 ***
## ---
## Signif. codes: 0 '***' 0.001 '**' 0.01 '*' 0.05 '.' 0.1 ' ' 1
##
## Residual standard error: 0.5502 on 12 degrees of freedom
## Multiple R-squared: 0.8772, Adjusted R-squared: 0.867
## F-statistic: 85.76 on 1 and 12 DF, p-value: 8.16e-07

#Mantis non-phylogenetic
m.mod<-lm(log10(H.prey.mass)~log10(Mass.mg),data=data.1)
summary(m.mod)

##
## Call:
## lm(formula = log10(H.prey.mass) ~ log10(Mass.mg), data = data.1)
##
## Residuals:
## Min 1Q Median 3Q Max
## -0.17860 -0.08533 -0.07193 0.05758 0.35689
##
## Coefficients:
## Estimate Std. Error t value Pr(>|t|)
## (Intercept) -1.2842 0.3485 -3.685 0.00312 **
## log10(Mass.mg) -0.8676 0.1147 -7.563 6.65e-06 ***
## ---
## Signif. codes: 0 '***' 0.001 '**' 0.01 '*' 0.05 '.' 0.1 ' ' 1
##
## Residual standard error: 0.1595 on 12 degrees of freedom
## Multiple R-squared: 0.8266, Adjusted R-squared: 0.8121
## F-statistic: 57.2 on 1 and 12 DF, p-value: 6.651e-06

confint(m.mod,level=0.95)

## 2.5 % 97.5 %
## (Intercept) -2.043415 -0.5249495
## log10(Mass.mg) -1.117528 -0.6176412

#insects
i.mod<-lm(log10(newdata$H.prey.mass)~log10(newdata$Predator.mass..mg.))
summary(i.mod)

##
## Call:
## lm(formula = log10(newdata$H.prey.mass) ~ log10(newdata$Predator.mass..mg.))
##
## Residuals:
## Min 1Q Median 3Q Max
## -6.4543 -0.6189 0.0177 0.6885 2.9634
##
## Coefficients:
## Estimate Std. Error t value Pr(>|t|)
## (Intercept) -0.3037 0.1635 -1.858 0.0649 .
## log10(newdata$Predator.mass..mg.) -0.7630 0.1107 -6.895 9.97e-11 ***
## ---
## Signif. codes: 0 '***' 0.001 '**' 0.01 '*' 0.05 '.' 0.1 ' ' 1
##
## Residual standard error: 1.469 on 171 degrees of freedom
## Multiple R-squared: 0.2175, Adjusted R-squared: 0.213
## F-statistic: 47.54 on 1 and 171 DF, p-value: 9.97e-11

confint(i.mod,level=0.95)

## 2.5 % 97.5 %
## (Intercept) -0.6264826 0.01898789
## log10(newdata$Predator.mass..mg.) -0.9814908 -0.54459187

#arachnids
a.mod<-lm(log10(Arach.data$H.prey.mass)~log10(Arach.data$Predator.mass..mg.))
summary(a.mod)

##
## Call:
## lm(formula = log10(Arach.data$H.prey.mass) ~ log10(Arach.data$Predator.mass..mg.))
##
## Residuals:
## Min 1Q Median 3Q Max
## -6.8216 -0.3704 0.2673 0.8210 3.0190
##
## Coefficients:
## Estimate Std. Error t value Pr(>|t|)
## (Intercept) -0.4306 0.2268 -1.899 0.0623 .
## log10(Arach.data$Predator.mass..mg.) -1.0624 0.1771 -5.998 1.18e-07 ***
## ---
## Signif. codes: 0 '***' 0.001 '**' 0.01 '*' 0.05 '.' 0.1 ' ' 1
##
## Residual standard error: 1.789 on 61 degrees of freedom
## Multiple R-squared: 0.371, Adjusted R-squared: 0.3607
## F-statistic: 35.98 on 1 and 61 DF, p-value: 1.177e-07

confint(a.mod,level=0.95)

## 2.5 % 97.5 %
## (Intercept) -0.884022 0.02288483
## log10(Arach.data$Predator.mass..mg.) -1.416569 -0.70821573

#Fishes
f.mod<-lm(log10(Fish.data$H.prey.mass)~log10(Fish.data$Predator.mass..mg.))
summary(f.mod)

##
## Call:
## lm(formula = log10(Fish.data$H.prey.mass) ~ log10(Fish.data$Predator.mass..mg.))
##
## Residuals:
## Min 1Q Median 3Q Max
## -7.1108 -0.7722 -0.2708 0.6916 4.1940
##
## Coefficients:
## Estimate Std. Error t value Pr(>|t|)
## (Intercept) -0.8688 0.4152 -2.092 0.0401 *
## log10(Fish.data$Predator.mass..mg.) -0.6897 0.1092 -6.313 2.28e-08 ***
## ---
## Signif. codes: 0 '***' 0.001 '**' 0.01 '*' 0.05 '.' 0.1 ' ' 1
##
## Residual standard error: 1.478 on 69 degrees of freedom
## Multiple R-squared: 0.3662, Adjusted R-squared: 0.357
## F-statistic: 39.86 on 1 and 69 DF, p-value: 2.278e-08

confint(f.mod,level=0.95)

## 2.5 % 97.5 %
## (Intercept) -1.6971921 -0.04040658
## log10(Fish.data$Predator.mass..mg.) -0.9076385 -0.47176426

#Mammals
#test if Sorex cinereus cinereus from regression as it is an outlier
library(outliers)

##
## Attaching package: 'outliers'

## The following object is masked from 'package:phytools':
##
## scores

grubbs.test(log10(Mamm.data$H.prey.mass))

##
## Grubbs test for one outlier
##
## data: log10(Mamm.data$H.prey.mass)
## G = 2.92286, U = 0.22875, p-value = 0.0005101
## alternative hypothesis: lowest value -13.3946622989108 is an outlier

outlier(log10(Mamm.data$H.prey.mass),logical=TRUE)

## [1] FALSE FALSE FALSE FALSE FALSE FALSE FALSE FALSE FALSE FALSE TRUE FALSE
## [13] FALSE

#yes significant outlier, remove from dataset
#keep in plot
Mamm.data.1<-Mamm.data[-11,]
mam.mod<-lm(log10(Mamm.data.1$H.prey.mass)~log10(Mamm.data.1$Predator.mass..mg.))
summary(mam.mod)

##
## Call:
## lm(formula = log10(Mamm.data.1$H.prey.mass) ~ log10(Mamm.data.1$Predator.mass..mg.))
##
## Residuals:
## Min 1Q Median 3Q Max
## -1.1704 -0.4751 -0.2937 0.5104 2.0268
##
## Coefficients:
## Estimate Std. Error t value Pr(>|t|)
## (Intercept) -1.6805 1.2914 -1.301 0.2223
## log10(Mamm.data.1$Predator.mass..mg.) -0.6286 0.2016 -3.118 0.0109 *
## ---
## Signif. codes: 0 '***' 0.001 '**' 0.01 '*' 0.05 '.' 0.1 ' ' 1
##
## Residual standard error: 0.9158 on 10 degrees of freedom
## Multiple R-squared: 0.4929, Adjusted R-squared: 0.4422
## F-statistic: 9.722 on 1 and 10 DF, p-value: 0.01091

confint(mam.mod,level=0.95)

## 2.5 % 97.5 %
## (Intercept) -4.557802 1.1968273
## log10(Mamm.data.1$Predator.mass..mg.) -1.077856 -0.1793998

## Figure 3A

#to save as a png use code below
#png(file="Ingestion time allometry predator mass h comp log10.png", height=10, width=10, units="in",res=300)
par(mar=c(5.1,6,4.1,2.1))

plot(log10(newdata$Predator.mass..mg.),log10(newdata$H.prey.mass),pch=21,bg=rgb(190/255,190/255,190/255,alpha=0.5),xlim=c(-1.7,9),ylim=c(-14,4), xlab=expression(log[10]~Predator~mass~(mg)),ylab=expression(log[10]~Handling~time/Prey~mass~(mg/days)),cex.lab=1.5,cex=1.5,axes=FALSE)
axis(1)
axis(2)

points(log10(Mol.data$Predator.mass..mg.),log10(Mol.data$H.prey.mass),pch=23,bg=rgb(255/255,20/255,147/255,alpha=0.5),cex=1.5)
points(log10(Rep.data$Predator.mass..mg.),log10(Rep.data$H.prey.mass),pch=24,bg=rgb(154/255,205/255,50/255,alpha=0.5),cex=1.5)
points(log10(Fish.data$Predator.mass..mg.),log10(Fish.data$H.prey.mass),pch=25,bg=rgb(0/255,205/255,205/255,alpha=0.5),cex=1.5)
points(log10(Mamm.data$Predator.mass..mg.),log10(Mamm.data$H.prey.mass),pch=21,bg=rgb(205/255,149/255,12/255,alpha=0.5),cex=1.5)
points(log10(Arach.data$Predator.mass..mg.),log10(Arach.data$H.prey.mass),pch=22,bg=rgb(0/255,0/255,0/255,alpha=0.5),cex=1.5)
points(log10(data.1$Mass.mg),log10(data.1$H.prey.mass),pch=data.1$sh,bg=data.1$col,cex=3)
points(log10(newdata[m,2]),log10(newdata[m,6]),pch=22,bg="blue",cex=3)#only mantis in dataset

#insect line
abline(a=i.mod$coefficients[1],b=i.mod$coefficients[2],col="gray",lwd=3)

#mantis non-phylogenetic
abline(a=m.mod$coefficients[1],b=m.mod$coefficients[2],col=fcol[2],lwd=3)#non-phylogenetic
#mantis phylogenetic coefficients from model above
abline(a=-1.03011,b=-0.94665,col=fcol[2],lwd=3,lty=2)
#arachnids
abline(a=a.mod$coefficients[1],b=a.mod$coefficients[2],lwd=3)
#Fishes
abline(a=f.mod$coefficients[1],b=f.mod$coefficients[2],col="cyan3",lwd=3)
#Mammals
abline(a=mam.mod$coefficients[1],b=mam.mod$coefficients[2],col="darkgoldenrod3",lwd=3)
points(-1.5,-10,pch=21,bg="gray")
text(-1.5,-10,"Insects",cex=0.7,pos=4)
points(-1.5,-10.5,pch=23,bg="deeppink")
text(-1.5,-10.5,"Mollusks",cex=0.7,pos=4)
points(-1.5,-11,pch=24,bg="yellowgreen")
text(-1.5,-11,"Reptiles",cex=0.7,pos=4)
points(-1.5,-11.5,pch=25,bg="cyan3")
text(-1.5,-11.5,"Fish",cex=0.7,pos=4)
points(-1.5,-12,pch=21,bg="darkgoldenrod3")
text(-1.5,-12,"Mammals",cex=0.7,pos=4)
points(-1.5,-12.5,pch=22,bg="black")
text(-1.5,-12.5,"Arachnids",cex=0.7,pos=4)


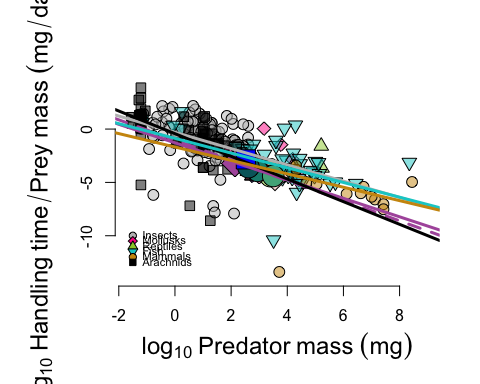


#dev.off()

## Figure 3B

Inset Figure 3B of mantis only data from above Put together in Adobe Illustrator

####plot of our data with confidence intervals and H. crassa
#To save as png use code below
#png(file="Ingestion time allometry predator mass H.prey log10.png", height=8, width=8, units="in",res=300)
par(mar=c(5.1,6,4.1,2.1))
plot(log10(data.1$Mass.mg),log10(data.1$H.prey.mass),pch=data.1$sh,bg=data.1$col,cex=4,xlab=expression(log[10]~Predator~Mass~(mg)),ylab=expression(log[10]~Handling~time/Prey~mass~(mg/days)),cex.lab=2,cex.axis=2,axes=FALSE,xlim=c(2,3.8),ylim=c(-4.8,-2))
Axis(side=1, labels=TRUE,cex.axis=2)
Axis(side=2, labels=TRUE,cex.axis=2)
mod.ht<-pgls(log10(H.prey.mass)~log10(Mass.mg) , df.phylo, lambda='ML')
summary(mod.ht)

##
## Call:
## pgls(formula = log10(H.prey.mass) ~ log10(Mass.mg), data = df.phylo,
## lambda = "ML")
##
## Residuals:
## Min 1Q Median 3Q Max
## -0.78564 -0.39663 -0.09593 0.05432 1.25996
##
## Branch length transformations:
##
## kappa [Fix] : 1.000
## lambda [ ML] : 1.000
## lower bound : 0.000, p = 0.067288
## upper bound : 1.000, p = 1
## 95.0% CI : (NA, NA)
## delta [Fix] : 1.000
##
## Coefficients:
## Estimate Std. Error t value Pr(>|t|)
## (Intercept) -1.03011 0.30215 -3.4093 0.00518 **
## log10(Mass.mg) -0.94665 0.10015 -9.4522 6.56e-07 ***
## ---
## Signif. codes: 0 '***' 0.001 '**' 0.01 '*' 0.05 '.' 0.1 ' ' 1
##
## Residual standard error: 0.5502 on 12 degrees of freedom
## Multiple R-squared: 0.8772, Adjusted R-squared: 0.867
## F-statistic: 85.76 on 1 and 12 DF, p-value: 8.16e-07

mod<-lm(log10(H.prey.mass)~log10(Mass.mg),data=data.1)
summary(mod)

##
## Call:
## lm(formula = log10(H.prey.mass) ~ log10(Mass.mg), data = data.1)
##
## Residuals:
## Min 1Q Median 3Q Max
## -0.17860 -0.08533 -0.07193 0.05758 0.35689
##
## Coefficients:
## Estimate Std. Error t value Pr(>|t|)
## (Intercept) -1.2842 0.3485 -3.685 0.00312 **
## log10(Mass.mg) -0.8676 0.1147 -7.563 6.65e-06 ***
## ---
## Signif. codes: 0 '***' 0.001 '**' 0.01 '*' 0.05 '.' 0.1 ' ' 1
##
## Residual standard error: 0.1595 on 12 degrees of freedom
## Multiple R-squared: 0.8266, Adjusted R-squared: 0.8121
## F-statistic: 57.2 on 1 and 12 DF, p-value: 6.651e-06

abline(a=mod$coefficients[1],b=mod$coefficients[2],col=fcol[2],lwd=3)#non-phylogenetic
abline(a=-1.03011,b=-0.94665,col=fcol[2],lwd=3,lty=2)
data.1$lg.time.h<-log10(data.1$H.prey.mass)
data.1$lg.mass.mg<-log10(data.1$Mass.mg)
pGLS_ci<-gls.ci(data.1$lg.time.h,data.1$lg.mass.mg,vcv(tree.1))#note run function below first
 lines(pGLS_ci$CI.plot$X,pGLS_ci$CI.plot$Lower5,lty=3,col=fcol[2],lwd=3)
 lines(pGLS_ci$CI.plot$X,pGLS_ci$CI.plot$Upper5,lty=3,col=fcol[2],lwd=3)
points(log10(newdata[m,2]),log10(newdata[m,6]),pch=22,bg="blue",cex=4)#only mantis in dataset


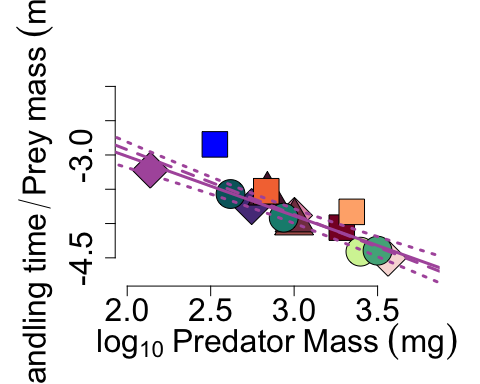


#dev.off()
